# The Effect of 12-Week e-Cigarette Use on Smoking Abstinence at 1 Year: The E3 Trial

**DOI:** 10.1016/j.jacadv.2025.101833

**Published:** 2025-06-25

**Authors:** Kristian B. Filion, Tetiana Zolotarova, Andréa Hébert-Losier, Sarah B. Windle, Pauline Reynier, Todd Greenspoon, Tim Brandys, Tamàs Fülöp, Thang Nguyen, Stéphane Elkouri, Igor Wilderman, Olivier F. Bertrand, Joanna Alexis Bostwick, Yves Lacasse, Smita Pakhale, Mark J. Eisenberg

**Affiliations:** a Departments of Medicine and of Epidemiology, Biostatistics and Occupational Health, McGill University, Quebec, Canada; b Faculty of Medicine, McGill University, Montreal, Quebec, Canada; c Centre for Clinical Epidemiology, Lady Davis Institute, Jewish General Hospital, Montreal, Quebec, Canada; d Family Medicine, McMaster University, Hamilton, Ontario, Canada; e Division of Vascular and Endovascular Surgery, Ottawa Hospital/University of Ottawa, Ottawa, Ontario, Canada; f Geriatric Division, Université de Sherbrooke, Sherbrooke, Quebec, Canada; g Department of Internal Medicine, University of Manitoba, Winnipeg, Manitoba, Canada; h Division of Vascular Surgery, Centre hospitalier de l’Université de Montréal, Montreal, Quebec, Canada; i Wilderman Medical Clinic, Thornhill, Ontario, Canada; j Institut Universitaire de Cardiologie et de Pneumologie de Québec (IUCPQ), Québec City, Quebec, Canada; k Department of Emergency Medicine, University of Ottawa, Ottawa, Ontario, Canada; l Multidisciplinary Department of Pulmonology and Thoracic Surgery, Institut universitaire de cardiologie et de pneumologie de Québec, Laval University, Montreal, Quebec, Canada; m Ottawa Hospital Research Institute, Ottawa, Ontario, Canada; n Division of Cardiology, Jewish General Hospital/McGill University, Montreal, Quebec, Canada

**Keywords:** smoking cessation, e-cigarettes, tobacco harm reduction, behavioral support, randomized controlled trial

## Abstract

**Background:**

The current evidence regarding the long-term efficacy of electronic cigarettes (e-cigarettes) for smoking cessation is unclear.

**Objectives:**

The purpose of this study was to assess the efficacy, safety, and tolerability of nicotine and non-nicotine e-cigarettes for smoking cessation in the general population.

**Methods:**

We randomized 376 adults who smoked ≥10 cigarettes/day and were motivated to quit at 17 Canadian sites to 12 weeks of nicotine (15 mg/mL) e-cigarettes (n = 128), non-nicotine e-cigarettes (n = 127), or no e-cigarettes (n = 121). All groups received individual counseling. The primary endpoint was point prevalence abstinence (7-day recall, biochemically validated using expired carbon monoxide) at 12 weeks. The 52-week follow-up results are reported here.

**Results:**

Participants (mean age 52 ± 13 years; 47% female) smoked a mean of 21 ± 11 cigarettes/day at baseline. Compared to individual counseling alone, participants randomized to nicotine e-cigarettes plus counseling had higher rates of point prevalence (23.6% vs 9.9%; difference: 13.7%; 95% CI: 4.6%-22.8%) and continuous abstinence (3.1% vs 0.0%; difference: 3.1%; 95% CI: 0.1%-6.2%) and greater reductions in the number of cigarettes smoked (−9.5 ± 10.5 vs −5.6 ± 9.5; difference: −3.9; 95% CI: −6.5 to −1.4) at 52 weeks. Benefits were also observed among participants randomized to non-nicotine e-cigarettes plus counseling vs counseling alone. No differences in abstinence or reduction were found between nicotine and non-nicotine e-cigarettes.

**Conclusions:**

Compared to individual counseling alone, short-term use of standardized nicotine and non-nicotine e-cigarettes plus counseling is efficacious at increasing smoking abstinence at 52 weeks.

Conventional combustible cigarettes remain the leading cause of preventable morbidity and mortality in North America, with 20% of U.S. adults continuing to smoke despite its documented health risks.^1^^,^^2^ More than one-quarter of individuals attempting to quit smoking combustible cigarettes now use electronic cigarettes (e-cigarettes) to manage their tobacco-dependence.^3^^,^^4^ However, the efficacy of e-cigarette use for smoking cessation is debated due to conflicting available evidence, and limited data available on its long-term effects.^5^^,^^6^ E3 (The Evaluating the Efficacy of e-Cigarette Use for Smoking Cessation) Trial^7^ was designed to address this knowledge gap. We previously reported increased smoking abstinence with e-cigarettes plus individual counseling vs counseling alone at 12 and 24 weeks.^8^ In this report, we present the final 1-year follow-up results of the E3 Trial.

## Methods

### Study design and population

E3 was a multicenter randomized controlled trial (RCT) conducted at 17 centers across Canada. Detailed information on the study design has been published elsewhere,^7^^,^^8^ and the trial protocol can be found in the Supplemental Appendix. The trial was approved by the research ethics boards of participating centers and conducted according to all institutional, provincial, and federal regulatory requirements. The trial was monitored by an external Data and Safety Monitoring Board (DSMB), which conferred before enrollment of the first participant and every 6 months thereafter.

We recruited participants through community-based advertisements (eg, fliers, newspaper), online platforms (Craigslist, Kijiji, and social media), as well as at clinics (outpatient, smoking cessation, and walk-in clinics). Preliminary screening for eligibility was conducted either in person or by phone. Individuals were eligible for inclusion if they were aged ≥18 years, actively smoked an average of more than 10 cigarettes/day over the last year and expressed a moderate or strong desire and intention to quit smoking (score ≥5 on the Motivation to Stop Scale).^9^

**Figure 1:**
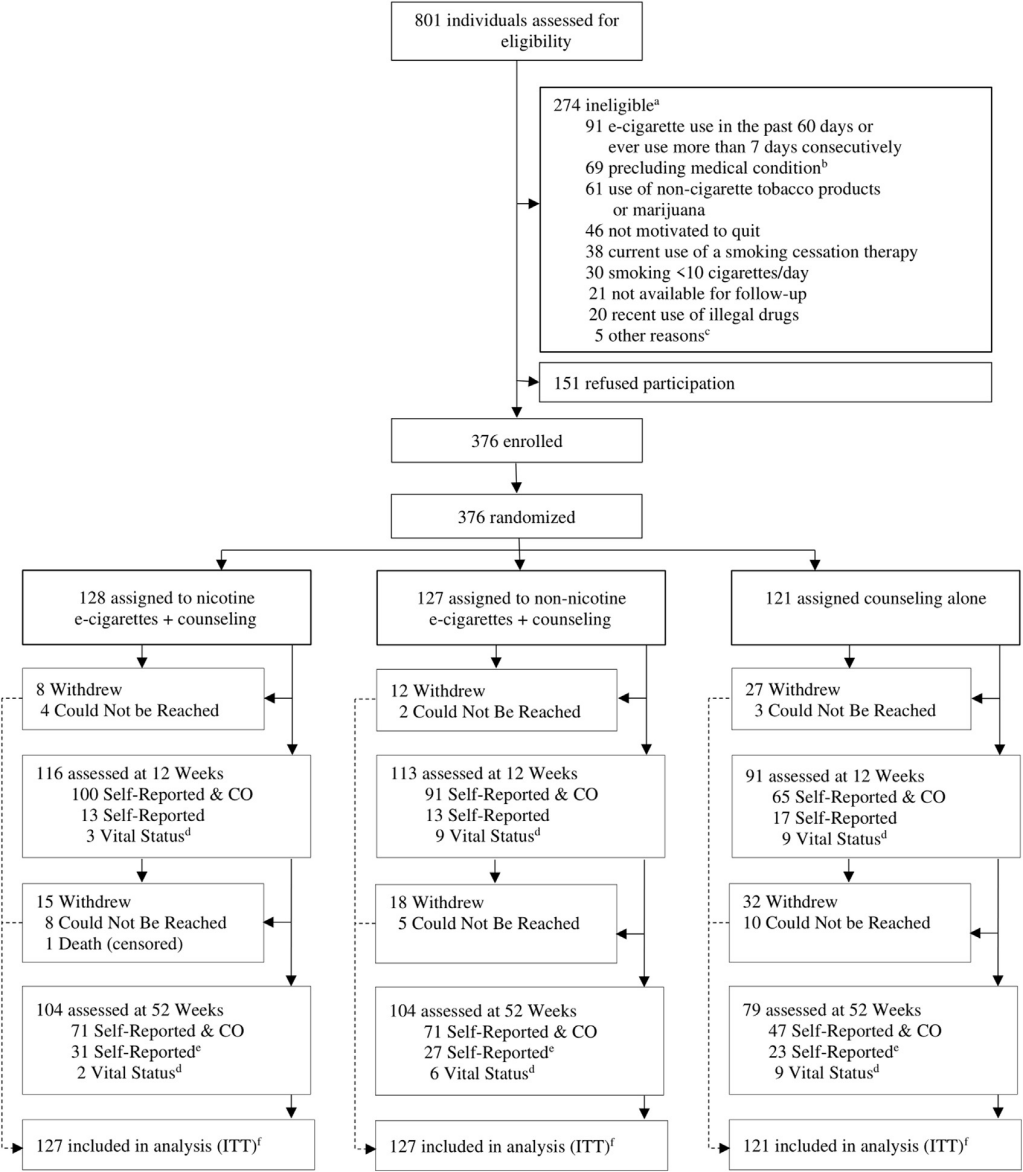
**Design of the E3 Trial of e-Cigarettes for Smoking Cessation** ^a^Subgroups sum >274, as screened individuals could have more than one reason. ^b^History of psychosis, schizophrenia, or bipolar disorder (n = 35; 6%); current cancer or in remission for <1 year (n = 22; 4%); condition with a prognosis of <1 year (n = 7; 1%); <1 month following a major cardiac event (n = 5; 1%). ^c^Pregnant/lactating female (n = 3; 0.5%); unable to provide informed consent in English or French (n = 2; 0.3%). ^d^For participants who were loss to follow-up, vital status was obtained from chart review or alternate contact. ^e^Due to COVID-19 pandemic restrictions, 17 (13%) in the nicotine e-cigarettes plus counseling, 12 (9%) in the non-nicotine e-cigarettes plus counseling, and 11 (9%) in the counseling-only groups completed their week 52 follow-up over the phone. ^f^Includes all participants except those who died. Participants who were lost to follow-up or withdrew were assumed to have returned to smoking at their baseline level.

Individuals were not eligible for inclusion if they had used a smoking cessation therapy in the past month, used an e-cigarette in the past 2 months, or ever used an e-cigarette for more than 7 days consecutively. Participants were also excluded if they had a history of mental health disorders (psychosis, schizophrenia, or bipolar disorder), current cancer or recent remission (<1 year), a condition with a prognosis of <1 year, experienced a major cardiovascular event (myocardial infarction, life-threatening arrhythmia, severe or worsening angina pectoris, or cerebral vascular accident) within the last month, used non-cigarette tobacco products, or smoked marijuana. Written informed consent was obtained for all participants by a noninvestigator research staff member at the baseline visit. Enrollment started in November 2016 across Canada (Figure 1). Due to a prolonged and unforeseen delay in the manufacturing of study e-cigarettes, enrollment was paused on September 27, 2019, and it was subsequently terminated on November 14, 2019, after consultation with the trial DSMB.^8^

Participants who were eligible for inclusion and provided consent were randomized 1:1:1 to one of three arms: 1) nicotine e-cigarette use plus individual counseling; 2) non-nicotine e-cigarette use plus individual counseling; or 3) individual counseling with no e-cigarette use. Individual counseling was selected as the comparator arm to ensure that all participants received evidence-based therapy while allowing for a clear assessment of the efficacy of e-cigarettes for smoking cessation. The non-nicotine e-cigarette use plus counseling arm was included to explore the behavioral aspect of e-cigarette use. Eligible participants were randomized via an online centralized system using a computer-generated randomization list. Randomization used permuted blocks of 6 and 9 and was stratified by study center. Participants, investigators, and study personnel were blinded to the nicotine content of the e-cigarettes.

## Interventions

Participants randomized to e-cigarettes (with or without nicotine) received a 12-week supply of e-cigarettes (Supplemental Figure 1) consisting of 21 tobacco-flavored liquid cartridges (15 or 0 mg nicotine/mL depending on the treatment arm) that were manufactured specifically for use in clinical studies (purchased from NJOY Inc). The e-cigarettes with and without nicotine were identical in appearance. Participants were asked to use their e-cigarettes as needed given their varying levels of nicotine dependence and individual habits. Additional cartridges were supplied as needed during the treatment period. No tapering schedule was specified in the protocol due to the variability in use among participants; however, participants were informed that they would have to return their e-cigarettes and unused cartridges following the 12-week treatment period.

The counseling focused on smoking cessation/relapse prevention and was provided by trained research personnel. The duration of the counseling was a minimum of 30 minutes at baseline, 10 minutes for telephone follow-ups, and 15 to 20 minutes at clinic follow-up visits. The following approaches were used: development/revision of a quit plan, encouragement of self-monitoring, review of triggers and challenges, and coping skills.^7^ Participants randomized to e-cigarettes also received counseling on adherence and challenges surrounding the use of e-cigarettes. Participants were not required to quit immediately at baseline; individualized quit plans allowed participants to gradually reduce conventional cigarette smoking before quitting if desired.

### Follow-up

Telephone follow-ups were conducted at weeks 1, 2, 8, and 18, and clinic visits were conducted at weeks 4, 12, 24, and 52. In this article, we report the 52-week follow-up results. Self-reported smoking (7-day recall), adherence, and adverse events (AEs) were assessed at each follow-up visit. Self-reported smoking abstinence was biochemically validated at clinic visits using exhaled carbon monoxide (eCO) ≤10 ppm (Micro 3 and 4 Smokerlyzer, Bedfont Scientific, Ltd).^10^ The following questionnaires were also completed by participants during clinic visits: the Glover Nilsson Smoking Behavioral Questionnaire (to assess behavioral dependence on smoking)^11^ and the Beck Depression Inventory (BDI-II) (to assess depressive symptoms).^12^ Nicotine dependence was assessed using the Fagerström Test for Nicotine Dependence at baseline.^13^

To limit losses to follow-up, we contacted the participants through several methods (eg, telephone, email, and mail) for up to 12 attempts for each follow-up. For visits that would otherwise not be completed (eg, participants unwilling/unable to complete follow-up), we requested minimal, high-priority data only (vital and smoking status and the occurrence of serious adverse events [SAEs]). Participants for which these minimal data were not collected were considered lost to follow-up.

### Endpoint assessment

The primary endpoint of the E3 Trial was point prevalence smoking abstinence at 12 weeks following randomization, defined as self-reported abstinence in the past 7 days with an eCO ≤10 ppm. The seven secondary endpoints were 1) point prevalence abstinence at other follow-ups; 2) continuous abstinence defined as self-reported abstinence at all follow-ups since baseline with an eCO ≤10 ppm at all clinic visits; 3) change in daily cigarette consumption from baseline at all follow-ups; 4) SAEs (adjudicated by an Endpoints Evaluation Committee); 5) AEs; 6) drop outs secondary to experiencing side effects; and 7) adherence to the assigned treatment.

### Power and sample size calculations

The planned sample of 162 participants per arm (total of 486) would have provided >80% power to detect a ≥12% absolute difference in abstinence at 52 weeks (the original primary endpoint), assuming an abstinence rate of 10% for counseling alone and a two-tailed alpha of 0.05. This treatment effect is consistent with previous trials of nicotine replacement therapies (NRTs).^14^ Due to early termination of enrollment, the study power was <68% at 52 weeks. Consequently, the timing of the primary endpoint measurement was changed to 12 weeks following consultation with the DSMB.^8^

### Statistical analyses

We used an intention-to-treat approach in which participants and outcomes were analyzed according to the assigned treatment arm regardless of the treatment received. If a participant withdrew from the study or was lost to follow-up, they were included in the intention-to-treat analysis and considered to have returned to smoking at their baseline level. Baseline characteristics are described with continuous data presented using the mean ± SD or median with 25th and 75th percentiles (Q1-Q3) and categorical data presented as count (percentage).

The primary comparison of this 52-week analysis was point prevalence abstinence at 52 weeks for nicotine e-cigarettes plus counseling vs counseling alone. Secondary analyses compared the other groups in pair-wise comparisons at all follow-ups. For each pair-wise comparison of binary outcomes, risk differences (RDs) with corresponding 95% CIs were estimated based on the binomial distribution. Cumulative incidence curves were plotted for continuous abstinence. For continuous outcomes, mean changes from baseline were estimated with corresponding 95% CIs. We assessed the distribution of change scores by visual inspection of histograms for each treatment group, which suggested approximate normality. Homogeneity of variance was tested using the Bartlett test, and the Welch method was used when variances were unequal. Prespecified secondary analyses as well as prespecified and post hoc sensitivity analyses are described in the Supplemental Methods. All statistical analyses were performed using SAS version 9.4 (SAS Institute, Inc). This trial is registered with ClinicalTrials.gov, NCT02417467.

## Results

From November 15, 2016, to September 27, 2019, 801 participants were screened for eligibility. A total of 376 individuals were enrolled in the trial (47.3% female). Participants were randomly assigned to the nicotine e-cigarette use plus counseling (n = 128), non-nicotine e-cigarette use plus counseling (n = 127), or counseling alone with no e-cigarette use (n = 121) arms (Figure 1). At baseline, participants had a mean age of 52 ± 13 years, smoked a mean of 21 ± 11 cigarettes/day, and had smoked for a mean of 34 ± 14 years. The study groups were generally balanced (Supplemental Table 1), with the exception of prior e-cigarette use: 43.0% in the nicotine group, 37.8% in non-nicotine group, and 27.3% in counseling alone group (Table 1). A total of 104 participants (81.3%) in the nicotine e-cigarette plus counseling group, 104 (81.9%) in the non-nicotine e-cigarette plus counseling group, and 79 (65.3%) in the counseling-alone group completed the 52-week follow-up (Figure 1). Self-reported smoking data were available at 52 weeks for 270 participants (71.8%) with eCO measurements obtained for 189 out of 270 participants (70.0%).

**Table 1:** Baseline Characteristics of Participants by Treatment Group in the E3 Trial

	Nicotine e-Cigarettes + Individual Counseling (n = 128)	Non-Nicotine e-Cigarettes + Individual Counseling (n = 127)	Individual Counseling Alone (n = 121)
Demographic characteristics			
Age, y	52 ± 12.9	51 ± 13.1	52 ± 11.5
Sex			
Male	63 (49.2%)	71 (55.9%)	64 (52.9%)
Female	65 (50.8%)	56 (44.1%)	57 (47.1%)
Self-reported race			
White	120 (93.7%)	111 (87.4%)	104 (86.0%)
Black	1 (0.8%)	7 (5.5%)	3 (2.5%)
Other^a^	7 (5.5%)	9 (7.1%)	14 (11.6%)
Education			
More than high school	80 (62.5%)	79 (62.2%)	74 (61.2%)
Smoking characteristics			
Years smoked	34 ± 14.0	34 ± 14.3	34 ± 12.9
Cigarettes/day at baseline	21 ± 9.1	21 ± 11.3	21 ± 11.2
Previously attempted to quit	116 (90.6%)	118 (92.9%)	108 (89.3%)
Number of serious attempts to quit	3 (2-5)	2.5 (2-4)	3 (2-5)
Previously used abstinence aids for smoking cessation^b^	101 (78.9%)	100 (78.7%)	99 (81.8%)
Previously tried an e-cigarette	55 (43.0%)	48 (37.8%)	33 (27.3%)
Other smoker(s) at home	40 (31.3%)	45 (35.4%)	36 (29.8%)
Other lifestyle characteristics			
Body mass index	n = 128	n = 127	n = 120
≥30 kg/m^2^	48 (37.5%)	47 (37.0%)	45 (37.5%)
Alcoholic drinks/week	n = 128	n = 127	n = 120
Mean ± SD	4 ± 6.4	4 ± 7.9	3 ± 5.4
Questionnaires			
Motivation to Stop Scale^c^	n = 128	n = 127	n = 121
Mean score	6.0 ± 0.8	6.1 ± 0.8	6.3 ± 0.8
5 (“I want to stop smoking and hope to soon”)	41 (32.0%)	38 (29.9%)	30 (24.8%)
6 (“I really want to stop smoking and intend to in the next 3 months”)	45 (35.2%)	39 (30.7%)	28 (23.1%)
7 (“I really want to stop smoking and intend to in the next month”)	42 (32.8%)	50 (39.4%)	63 (52.1%)
Fagerström Test for Nicotine Dependence^d^	n = 128	n = 127	n = 119
Mean score	5.8 ± 2.1	5.6 ± 2.2	5.7 ± 2.2
Mild	19 (14.8%)	25 (19.7%)	21 (17.6%)
Moderate	60 (46.9%)	57 (44.9%)	54 (45.4%)
Severe	49 (38.3%)	45 (35.4%)	44 (37.0%)
Glover-Nilsson Smoking Behavioral Questionnaire^e^	n = 128	n = 126	n = 119
Mean score	21.1 ± 8.3	20.1 ± 8.2	20.2 ± 8.1
Mild	17 (13.3%)	25 (19.8%)	22 (18.5%)
Moderate	59 (46.1%)	55 (43.7%)	53 (44.5%)
Strong	43 (33.6%)	36 (28.6%)	33 (27.7%)
Very strong	9 (7.0%)	10 (7.9%)	11 (9.2%)
Beck Depression Inventory-II^f^	n = 127	n = 127	n = 118
Mean score	11.2 ± 9.2	9.7 ± 9.0	11.3 ± 9.6
Minimal	86 (67.7%)	92 (72.4%)	78 (66.1%)
Mild	19 (15.0%)	19 (15.0%)	18 (15.3%)
Moderate	15 (11.8%)	12 (9.4%)	14 (11.9%)
Severe	7 (5.5%)	4 (3.2%)	8 (6.8%)
Medical history^g^			
Cancer	11 (8.6%)	12 (9.5%)	14 (11.6%)
Depression^h^	45 (35.2%)	42 (33.1%)	36 (29.8%)
Diabetes	16 (12.8%)	24 (18.9%)	22 (18.2%)
Elevated cholesterol	47 (36.7%)	50 (39.4%)	46 (38.0%)
Heart disease	23 (18.0%)	22 (17.3%)	23 (19.0%)
Hypertension	41 (32.0%)	41 (32.3%)	33 (27.3%)
Respiratory problems	31 (24.2%)	40 (31.5%)	34 (28.1%)
Asthma	14 (10.9%)	18 (14.2%)	20 (16.5%)
Chronic obstructive pulmonary disease	13 (10.2%)	14 (11.0%)	11 (9.1%)
Chronic bronchitis	10 (7.8%)	10 (7.9%)	10 (8.3%)
Emphysema	5 (3.9%)	5 (3.9%)	5 (4.1%)
Other^i^	2 (1.6%)	5 (3.9%)	4 (3.3%)
More than one respiratory problem	11 (8.6%)	9 (7.1%)	11 (9.1%)

Values are n (%), mean ± SD, or median (Q1-Q3).

a Participants were asked to select “White,” “Black, ” or “other, specify: ”. Self-reported “other” includes: Israeli, Indigenous, Asian, Pilipino, Urdu, Italian, Arab, Trinidadian, Moroccan, Nepalese, Spanish, Tunisian, East Indian.

b Previously used abstinence aids includes: varenicline, bupropion, nicotine patch, nicotine gum, nicotine inhaler, nicotine lozenge, Nicotine QuickMist, counseling, other aids (acupuncture, hypnosis, laser, apps).

c Motivation to Stop Scale: Possible scores range between 1 and 7, with higher scores indicating stronger motivation to quit smoking. Potential participants completed this 1-item scale during screening and must have selected level 5 or higher to be eligible for the trial, indicating a moderate or strong desire and intention to attempt to quit.

d Fagerström Test for Nicotine Dependence: Possible scores range between 0 and 10, with higher scores indicating a stronger dependence on nicotine. Mild: 0 to 3; moderate: 4 to 6; severe: ≥7.

e Glover-Nilsson Smoking Behavioral Questionnaire: Possible scores range between 0 and 44, with higher scores indicating greater behavioral dependence on smoking. Mild: 0 to 12; moderate: 12 to 22; strong: 12 to 33; very strong: ≥34.

f Beck Depression Inventory-II: Possible scores range between 0 and 63, with higher scores indicating greater depressive symptoms. Minimal: 0 to 13; mild: 14 to 19; moderate: 20 to 28; severe: ≥29.

g Medical history was self-reported.

h Defined as prior use of medication for depression.

i Other (respiratory problems) includes: chronic pneumonia, shortness of breath, and sleep apnea.

**Figure 2:**
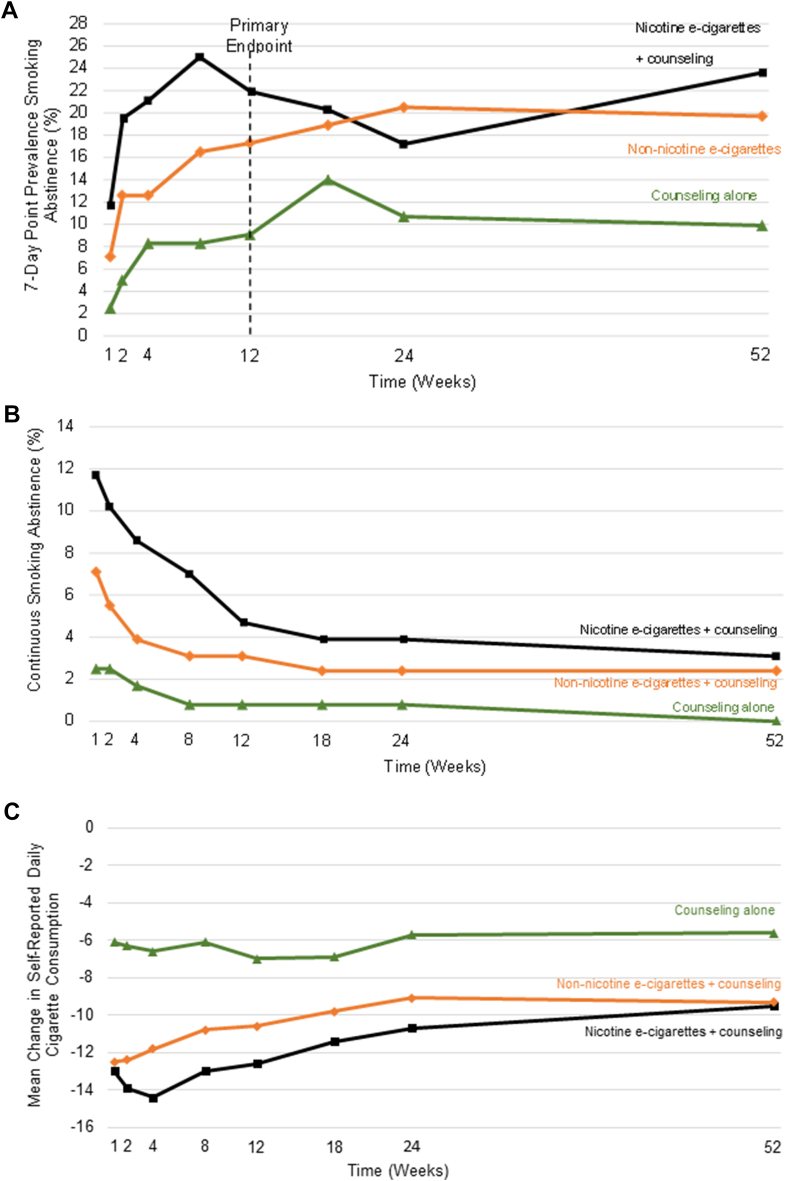
**Smoking Abstinence and Reduction by Treatment Group in the E3 Trial** (A) Point prevalence smoking abstinence, (B) Continuous smoking abstinence, (C) Change in self-reported daily cigarette consumption from baseline.

We previously reported that at 12 weeks, participants randomized to nicotine e-cigarettes with counseling achieved higher 7-day point prevalence abstinence rates compared to those randomized to counseling alone (21.9% vs 9.1%; RD: 12.8%; 95% CI: 4.0%-21.6%).^8^ At 52 weeks, similar differences in point prevalence abstinence were observed (Figure 2A, Supplemental Table 2). Participants randomized to nicotine e-cigarettes had a 7-day point prevalence abstinence at 52 weeks that was more than double that of participants randomized to counseling alone (23.6% vs 9.9%; RD: 13.7%; 95% CI: 4.6%-22.8%). Moreover, participants randomized to the non-nicotine e-cigarette arm also had higher 7-day point prevalence abstinence at 52 weeks compared to those randomized to counseling alone (19.7% vs 9.9%; RD: 9.8%; 95% CI: 1.0%-18.5%). There was no difference in point prevalence abstinence between participants in nicotine and non-nicotine e-cigarette groups at 52 weeks (23.6% vs 19.7%; RD: 3.9%; 95% CI: −6.2% to 14.1%).

Continuous abstinence remained consistently low throughout follow-up, with proportions below 4.0% in all treatment groups (Figure 2B, Supplemental Table 3). At 52 weeks, randomization to nicotine e-cigarettes resulted in a 3.1% (3.1% vs 0.0%; RD: 3.1%; 95% CI: 0.1%-6.2%) absolute difference in continuous abstinence compared to counseling alone. No difference was observed between either non-nicotine e-cigarettes vs counseling alone (2.4% vs 0%; RD: 2.4%; 95% CI: −0.3% to 5.0%) or between nicotine and non-nicotine e-cigarettes (3.1% vs 2.4%; RD: 0.8%; 95% CI: −3.2% to 4.8%).

Figure 2C describes the change in mean self-reported daily cigarette consumption in all 3 treatment groups. In both e-cigarette groups, daily cigarette consumption decreased rapidly after randomization, but the benefits diminished over time (Supplemental Table 4). At 52 weeks, both e-cigarette groups smoked a mean of 9 ± 11 or 12 cigarettes/day less than at baseline, while the counseling group smoked a mean of 6 ± 9 fewer cigarettes/day than at baseline (difference for nicotine e-cigarette vs counseling: −3.9; 95% CI: −6.5 to −1.4; difference for non-nicotine e-cigarettes vs counseling: −3.7; 95% CI: −6.4 to −1.0). No differences were observed between nicotine and non-nicotine e-cigarette arms in changes in cigarette consumption during the 52-week follow-up (−0.2; 95% CI: −3.0 to 2.6).

The utilization of nonstudy smoking cessation therapies increased in all groups between the 12-, 24- and 52-week follow-ups (Table 2). The most prominent change was observed in the nicotine e-cigarettes with counseling group, with a prevalence of 51.0% at 52 weeks, compared to 10.8% at 12 weeks, and 41.0% at 24 weeks. At 12 weeks, the prevalence of using nonstudy smoking cessation therapies was relatively higher in both the non-nicotine e-cigarette group (12.9%) and the counseling-only group (21.7%) compared to the nicotine e-cigarette group. However, these rates remained stable after the 24-week follow-up (34.9% in the non-nicotine e-cigarette group and 35.6% in the counseling-only group) and persisted similarly at 52 weeks (34.1% and 35.9%, respectively). The most frequently employed nonstudy-related smoking cessation therapy was nonstudy e-cigarettes, with a higher prevalence of use in the nicotine e-cigarette group compared to the non-nicotine and counseling-only groups (42.2% in the nicotine e-cigarette group vs 26.8% and 19.8% in the non-nicotine and counseling-only groups, respectively). Other frequently used nonstudy-related smoking cessation methods included NRT in the form of gum or patches (Table 3).

**Table 2:** The Frequency of Use of Nonstudy Smoking Cessation Therapy Reported at Follow-Up Visits by Treatment Group in the E3 Trial

Follow-Up	Nicotine e-Cigarettes + Individual Counseling	Non-Nicotine e-Cigarettes + Individual Counseling	Individual Counseling Alone
Wk 1	2/117 (1.7%)	3/122 (2.5%)	11/92 (12.0%)
Wk 2	3/118 (2.5%)	5/117 (4.3%)	11/85 (12.9%)
Wk 4	2/117 (1.7%)	5/114 (4.4%)	12/78 (15.4%)
Wk 8	4/108 (3.7%)	10/102 (9.8%)	11/68 (16.2%)
Wk 12	12/111 (10.8%)	13/101 (12.9%)	15/69 (21.7%)
Wk 18	43/102 (42.2%)	27/91 (29.7%)	25/68 (36.8%)
Wk 24	41/100 (41.0%)	30/86 (34.9%)	21/59 (35.6%)
Wk 52	49/96 (51.0%)	30/88 (34.1%)	23/64 (35.9%)

Descriptive statistics represent the number of participants who used nonstudy smoking cessation therapy over the total number of participants with available data (n/N), expressed as a percentage (%).

**Table 3:** The Types of Nonstudy Smoking Cessation Therapies Used During the 52 Weeks by Treatment Group in the E3 Trial^a^

	Nicotine e-Cigarettes + Counseling (n = 128)	Non-Nicotine e-Cigarettes + Counseling (n = 127)	Counseling Alone (n = 121)
Participants reporting any use	76 (59.4%)	54 (42.5%)	49 (40.5%)
Nonstudy e-cigarette	54 (42.2%)	34 (26.8%)	24 (19.8%)
Nicotine e-cigarette	36 (28.1%)	24 (18.9%)	17 (14.0%)
Non-nicotine e-cigarette	15 (11.7%)	7 (5.5%)	6 (5.0%)
Unknown e-cigarette nicotine content	10 (7.8%)	5 (3.9%)	4 (3.3%)
Nicotine replacement therapy	33 (25.8%)	20 (15.7%)	24 (19.8%)
Gum	17 (13.3%)	10 (7.9%)	12 (9.9%)
Patch	22 (17.2%)	7 (5.5%)	12 (9.9%)
QuickMist	4 (3.1%)	2 (1.6%)	2 (1.7%)
Inhaler	3 (2.3%)	2 (1.6%)	3 (2.5%)
Lozenge	3 (2.3%)	2 (1.6%)	0 (0.0%)
Non-nicotine replacement pharmaceuticals	3 (2.3%)	12 (9.4%)	9 (7.4%)
Varenicline	2 (1.6%)	8 (6.3%)	6 (5.0%)
Bupropion	1 (0.8%)	4 (3.1%)	3 (2.5%)
Counseling	5 (3.9%)	2 (1.6%)	2 (1.7%)
Other^b^	5 (3.9%)	8 (6.3%)	6 (5.0%)

a The denominator used to calculate percentages is the total number of participants randomized to each arm. Only the first use of each therapy was counted for each participant in each category. Percentages may sum to >100, as some participants used more than one therapy.

b Includes reported use of Cravv, hypnosis, laser therapy, marijuana, nicotine pills, phone apps, smoking cessation books, Snus, and rehabilitation.

At the 52-week follow-up, AEs were frequently observed among the 376 participants (Supplemental Tables 5 and 6). Most AEs were nonserious and of mild-to-moderate severity, with common incidents including cough (289, 76.9%), dry mouth (237, 63.0%), rhinitis (255, 67.8%), and headache (237, 63.0%), in line with our findings at the 12- and 24-week follow-ups.^8^ The number and proportion of AEs were comparable between the study groups with the slight prevalence in the e-cigarette groups when compared to the counseling alone group.

A total of 18 SAEs were reported among the study participants between the 24- and 52-week follow-up visits. Of these, 7 (5.5%) events occurred in the nicotine e-cigarettes plus counseling group, 6 (4.7%) events in the non-nicotine e-cigarettes plus counseling group, and 5 (4.1%) events in the counseling alone group (Supplemental Table 7). No SAE was determined to be related to the treatment.

**Figure 3:**
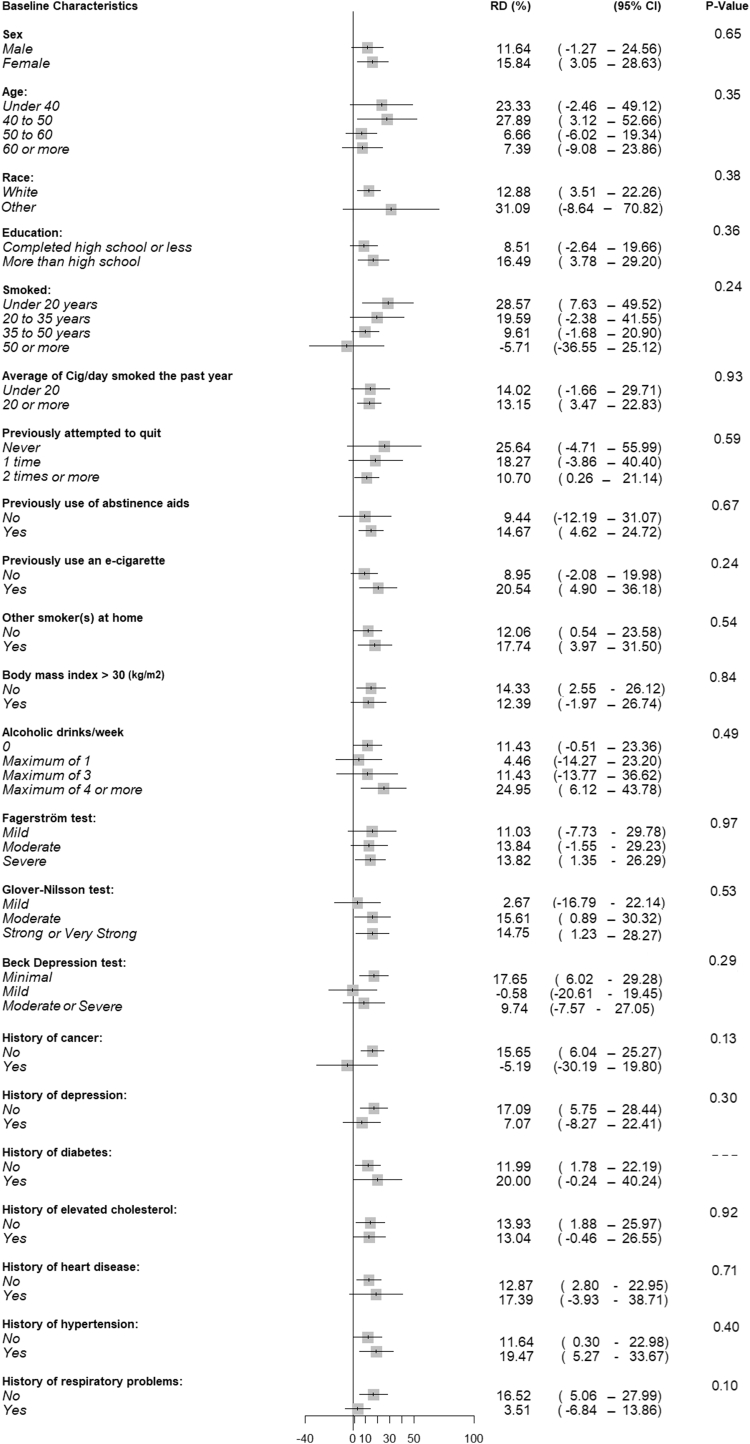
**Risk Differences for 7-Day Point Prevalence Smoking Abstinence at 52 Weeks Between Treatment Groups by Baseline Characteristics** Nicotine e-cigarettes with individual counseling vs individual counseling alone.

A secondary analysis examining effect measure modification was conducted but was underpowered to detect differences between groups (Figure 3, Supplemental Tables 8 and 9, Supplemental Figure 3).

We conducted prespecified sensitivity analyses to assess the impact of the assumption that participants who withdrew or were lost to follow-up had resumed smoking. These analyses encompassed both a complete case analysis and the use of multiple imputation to impute missing data on smoking abstinence and reduction (Supplemental Tables 2 to 4). The results of these sensitivity analyses revealed that point estimates were attenuated, and the 95% CIs were wider, underscoring that results were sensitive to missing data assumptions. To assess the impact of imbalances in baseline participant characteristics between groups, we constructed a prespecified logistic regression model at 52 weeks to adjust for baseline characteristics with an absolute value of the standardized difference of 0.1 or greater. Notably, these results were consistent with those of our primary analyses (Supplemental Table 10).

In post hoc analyses, we adjusted for the baseline value of cigarettes smoked per day; these results did not meaningfully change our treatment estimates or conclusions (Supplemental Table 11). We also assessed the impact of clustering by site using generalized linear mixed models, which incorporated site as a random effect; these results were similar to those of our main analyses (Supplemental Table 12). In Supplemental Figure 2, the results display a composite of participants who were categorized as abstinent or who had successfully reduced their daily cigarette consumption by 50% or more during clinic visits. The prevalence of abstinence or a ≥50% reduction reached a plateau and remained relatively consistent up to the 52 weeks in all 3 groups (Supplemental Table 13).

## Discussion

The E3 Trial was designed to evaluate the efficacy of nicotine and non-nicotine e-cigarettes, when used with individual counseling for smoking cessation, in comparison to counseling alone. We found that 12 weeks of nicotine or non-nicotine e-cigarettes, when compared to counseling alone, lead to an increase in point prevalence abstinence at 52 weeks among adults motivated to quit cigarette smoking. Furthermore, the groups receiving nicotine or non-nicotine e-cigarettes had higher rates of continuous abstinence and greater reductions in cigarette consumption compared to the counseling-only group at the same time point. Our results showed that even short-term use of e-cigarettes leads to greater smoking cessation at 52 weeks compared to counseling alone.

e-Cigarettes are a relatively recent method for tobacco dependence management, and the available evidence continues to expand. The efficacy of e-cigarettes at promoting smoking cessation appeared comparable to the U.S. Food and Drug Administration-approved varenicline, a recognized first-line pharmacological approach.^15^^,^^16^ Our findings are consistent with a 2024 Cochrane review of 88 studies (27,235 participants, 47 RCTs), which found that nicotine e-cigarette use for smoking cessation almost doubles quit rates at 6 months to 1 year when compared to NRT or behavioral support alone.^17^ Two subsequent RCTs published in 2024 further support our results and underscore the growing interest in and the critical relevance of this research area.^18^^,^^19^ Future research should prioritize investigating variations in e-cigarette efficacy across participant subgroups and identifying factors associated with sustained long-term smoking abstinence.

e-Cigarettes are proposed as a smoking cessation aid for certain individuals, yet concerns remain regarding the long-term benefits associated with their use.^17^ First-line smoking cessation therapies,^16^ including varenicline, bupropion, and NRT, are valuable but not a universal solution for people who smoke. A substantial proportion of people who have tried them continue to smoke despite their use.^20^^,^^21^ Only a limited number of RCTs have examined the efficacy of e-cigarettes for smoking cessation over a follow-up duration exceeding 8 months.^22^, ^23^, ^24^ The results of the E3 Trial contribute to the evidence in this area, particularly regarding whether using e-cigarettes for a short period of 12 weeks can lead to 12 months cessation of conventional cigarette smoking. This timeline aligns with studies assessing the long-term efficacy of first-line smoking cessation medications, where the treatment duration typically lasted 8 to 12 weeks and the reported follow-up period included 12 months.^20^^,^^21^ Our findings suggest that e-cigarettes could potentially serve as an effective alternative or supplementary intervention for individuals who have not achieved success with conventional cessation methods.

The notable increase in quit rates among participants in the e-cigarette groups at 12 months suggests that factors beyond the study’s prescribed interventions may have played some role. Research indicates that individuals who successfully quit smoking with the aid of e-cigarettes may use them for a more prolonged duration when compared to other smoking cessation pharmacotherapies.^22^^,^^24^ Almost half of the participants in the nicotine e-cigarette group in the E3 Trial reported use of nonstudy-related e-cigarette at the 52-week follow-up. However, a high proportion of participants in the E3 Trial used non-nicotine e-cigarettes, suggesting that these products may be beneficial for long-term smoking cessation. e-Cigarettes might possess the ability to replace the psychosocial, psychological, and social elements that individuals once derived satisfaction from with traditional smoking.^25^ Consequently, prolonged use of non-nicotine e-cigarettes may represent a less harmful approach for some individuals to become free from nicotine and tobacco smoking.

Continued safety concerns surround e-cigarettes, prompting the recommendation that their use for smoking cessation should be limited to short-term use. The 2024 Cochrane review suggests that there are no distinct disparities in SAEs between nicotine e-cigarettes and pharmacological interventions when compared to no pharmacotherapy or placebo.^15^ Our results indicated a low risk of SAEs that was similar between the interventional groups and the counseling alone group. While the long-term consequences of e-cigarette use remain uncertain, there is a consensus among experts that e-cigarettes are probably a safer option compared to conventional cigarettes.^26^ Whether serving as a bridge to smoking cessation or as a harm reduction tool for long-term use, e-cigarettes shield users from the toxicants generated by combustion, thus mitigating tobacco-related health risks. Results from a long-term prospective evaluation of respiratory parameters indicate that individuals with chronic obstructive pulmonary disease who use e-cigarettes and either quit smoking or reduce their cigarette consumption experience improvements in both quality of life and respiratory outcomes.^27^

Nicotine e-cigarettes have been proposed as a harm reduction tool for smokers, but their vascular effects remain debated. A study of 145 participants without cardiovascular disease showed significant vascular improvements within 1 month of switching to e-cigarettes, including better endothelial function and reduced arterial stiffness.^28^ While e-cigarettes may acutely affect arterial elasticity and oxidative stress, they have been linked to lower central and brachial systolic blood pressure, arterial wave reflections, and oxidative stress after a month.^29^^,^^30^ However, potential risks related to e-liquids cannot be disregarded, which can induce oxidative stress and form adducts with proteins, RNA, and DNA, ultimately impairing cellular function.^31^^,^^32^ Furthermore, nicotine itself is a vasoactive substance that stimulates inflammatory pathways and activates the sympathetic nervous system. Studies indicate that nicotine is associated with increased heart rate (10-15 beats/min) and systolic blood pressure (5-10 mm Hg).^33^ Future research may focus on the long-term impact of e-cigarettes on the vascular system and elucidate the underlying mechanisms.

e-Cigarettes, in contrast to varenicline or bupropion, are commercially available consumer products that require no monitoring from a physician in most jurisdictions. At the population level, the unrestricted availability of e-cigarettes carries inherent risks, as they can potentially act as a gateway to traditional smoking, particularly among young people. Unlike adults, most teenagers and young adults do not use e-cigarettes for quitting smoking.^4^ In 2021, Australian government reinforced its e-cigarette regulations, making all nicotine-containing e-cigarettes prescription-only medicines.^34^ This framework aims to prevent youth initiation while facilitating access for individuals who smoke seeking cessation with medical guidance. The efficacy of this regulatory tactic is yet to be determined.

Our trial had several potential limitations. First, the trial experienced premature termination, resulting in the recruitment of only 77% of the intended sample size. Second, participants in the E3 Trial were required to be motivated to quit smoking. Motivated individuals may have lower levels of nicotine dependence and, as a result, lower relapse rates. Third, eCO validation can only detect smoking within the last 24 hours, which may result in some false negative results. Fourth, we utilized standardized products specifically for use in clinical studies, which may introduce a potential source of bias and affect the the generalizability of the results. Fifth, the trial experienced a differential dropout rate among participants, and results were sensitive to missing data assumptions. Sixth, we did not adjust for multiple comparisons, increasing the likelihood of a chance finding; therefore, results should be interpreted with caution. Finally, while not explored in the present study, future research specifically designed to evaluate the impact of e-cigarette use intensity on smoking cessation is needed.

## Conclusions

Compared to individual counseling alone, short-term use of nicotine and non-nicotine e-cigarettes plus counseling is efficacious at increasing smoking abstinence at 52 weeks (Central Illustration). Similar benefits were observed between nicotine and non-nicotine e-cigarettes. The E3 Trial provides promising evidence regarding the efficacy of e-cigarettes when used for smoking cessation in a general population of adults who smoke.

Perspectives **COMPETENCY IN PATIENT CARE:** The E3 Trial shows short-term e-cigarette use can promote long-term smoking abstinence with minimal adverse events. Nicotine and non-nicotine e-cigarettes demonstrate varying efficacy, highlighting both pharmacological and behavioral impacts.

**TRANSLATIONAL OUTLOOK:** Future research should explore long-term efficacy, safety, and patient-centered strategies for e-cigarette use.

**Central Illustration:**
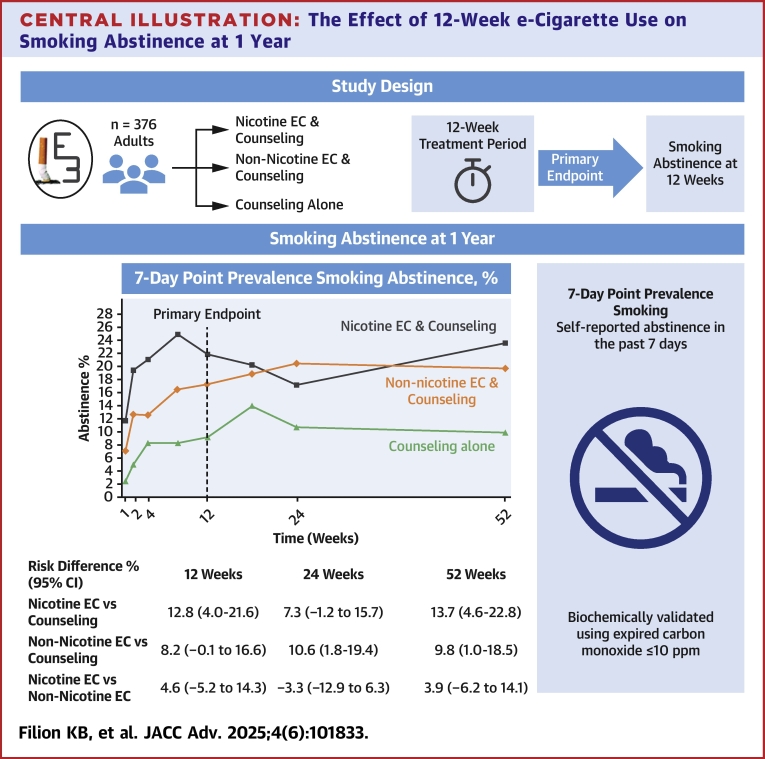
**The Effect of 12-Week e-Cigarette Use on Smoking Abstinence at 1 Year** EC = electronic cigarette; ppm = parts per million.

## Funding support and author disclosures

This study was funded by the Canadian Institutes of Health Research (CIHR, Canada) under grant numbers 133727 and 155969. Both nicotine and non-nicotine e-cigarettes were purchased from NJOY Inc (Scottsdale, AZ). The funder had no role in the design and conduct of the study; collection, management, analysis, or interpretation of the data; preparation, review, or approval of the manuscript; or the decision to submit the manuscript for publication. Dr Filion is supported by a William Dawson Scholar award from McGill University. Dr Windle is supported by a Canada Graduate Scholarship in Honour of Nelson Mandela from CIHR and a Doctoral Training Award from the Fonds de recherche du Québec–Santé. Dr Eisenberg is supported by a James McGill Professorship from McGill University. Dr Hébert-Losier conducted this work while employed at the Lady Davis Institute and is now an employee of Biospective Inc. All other authors have reported no relationships relevant to the contents of this paper to disclose.
