# Supplementary material for: The Effect of 12-Week e-Cigarette Use on Smoking Abstinence at 1 Year: The E3 Trial
Source: JACC Adv. 2025 Jun 25;4(6):101833. doi: 10.1016/j.jacadv.2025.101833 (PMC12277599; doi:10.1016/j.jacadv.2025.101833)
Supplement: Supplemental Data [file mmc1.docx]

**SUPPLEMENTAL APPENDIX**

**Supplemental Methods**

Pre-specified secondary, sensitivity and post-hoc analyses

In secondary analyses, we examined the effect of treatment group on daily cigarette consumption using linear regression. Multiple predetermined sensitivity analyses were performed. To challenge our assumption that participants who withdrew or were lost-to-follow-up returned to smoking, we conducted a complete case analysis (where only those with complete follow-up were included) and multiple imputation to impute missing outcome data. The process of multiple imputation was carried out using the completely conditional specification method, generating 5 imputed datasets. The findings were then integrated using Rubin’s principles.^1^ In addition, sensitivity analyses were performed to investigate the impact of imbalances in participants’ initial characteristics, which were characterized as having a standardized difference with an absolute value of ≥ 0.1. The study incorporated unbalanced participant characteristics as covariates in a logistic regression model to calculate the adjusted odds ratios (ORs) and 95% confidence intervals (CIs) for point prevalence abstinence at 12, 24, and 52 weeks.

Three post-hoc analyses were performed. To investigate the possibility of clustering by site, we employed generalized linear mixed models with a random-effect for site. This allowed us to estimate ORs and 95% CIs for point prevalence abstinence at 12, 24, and 52 weeks. A comparison was made between the baseline characteristics of participants who provided self-reported smoking data at 52 weeks and those who did not. Finally, to assess the impact of participants gradually discontinuing the trial during the first few weeks, we repeated our analyses of continuous abstinence excluding follow-up data from weeks 1 and 2.

**Supplemental Table 1:** Balance of baseline characteristics of participants by treatment group

|  | Standardized Differences | | |
| --- | --- | --- | --- |
| Characteristic | Nicotine  E-Cigarettes + Individual Counseling  vs  Individual Counseling Alone | Nicotine  E-Cigarettes + Individual Counseling  vs  Non-Nicotine E-Cigarettes + Individual Counseling | Non-Nicotine E-Cigarettes + Individual Counseling  vs  Individual Counseling Alone |
| Demographic Characteristics |  |  |  |
| Age (mean years) | 0.01 | 0.05 | 0.05 |
| Sex |  |  |  |
| Male | 0.07 | 0.13 | 0.6 |
| Female | 0.07 | 0.13 | 0.6 |
| Self-Reported Race |  |  |  |
| White | 0.26 | 0.22 | 0.4 |
| Black | 0.13 | 0.27 | 0.16 |
| Other^a^ | 0.22 | 0.07 | 0.15 |
| Education |  |  |  |
| More than high school | 0.03 | 0.01 | 0.02 |
| Smoking Characteristics |  |  |  |
| Years smoked (mean) | 0.03 | 0.02 | 0.01 |
| Cigarettes/day at baseline (mean) | 0.07 | 0.07 | 0.00 |
| Previously attempted to quit | 0.05 | 0.08 | 0.13 |
| Number of serious attempts to quit (median) |  |  |  |
| Previously used abstinence aids^b^ for smoking cessation | 0.07 | 0.00 | 0.08 |
| Previously tried an e-cigarette | 0.33 | 0.11 | 0.23 |
| Other smoker(s) at home | 0.03 | 0.09 | 0.12 |
| Other Lifestyle Characteristics |  |  |  |
| Body Mass Index ≥ 30 kg/m^2^ | 0.00 | 0.01 | 0.01 |
| Alcoholic drinks/week (mean) | 0.03 | 0.05 | 0.08 |
| Questionnaires | | | |
| Motivation to Stop Scale^c^ | n=128 | n=127 | n=121 |
| Mean Score | 0.32 | 0.11 | 0.21 |
| 5 (‘I want to stop smoking and hope to soon’) | 0.16 | 0.05 | 0.12 |
| 6 (‘I really want to stop smoking and intend to in the next 3 months’) | 0.27 | 0.09 | 0.17 |
| 7 (‘I really want to stop smoking and intend to in the next month’) | 0.40 | 0.14 | 0.26 |
| Fagerström Test for Nicotine Dependence^d^ | n=128 | n=127 | n=119 |
| Mean Score | 0.07 | 0.10 | 0.03 |
| Mild | 0.08 | 0.13 | 0.05 |
| Moderate | 0.03 | 0.04 | 0.05 |
| Severe | 0.03 | 0.06 | 0.03 |
| Glover-Nilsson Smoking Behavioral Questionnaire^e^ | n=128 | n=126 | n=119 |
| Mean Score | 0.12 | 0.12 | 0.00 |
| Mild | 0.14 | 0.18 | 0.03 |
| Moderate | 0.03 | 0.05 | 0.02 |
| Strong | 0.13 | 0.11 | 0.02 |
| Very Strong | 0.08 | 0.03 | 0.05 |
| Beck Depression Inventory-II^f^ | n=127 | n=127 | n=118 |
| Mean Score | 0.01 | 0.17 | 0.18 |
| Minimal | 0.03 | 0.10 | 0.14 |
| Mild | 0.01 | 0.00 | 0.01 |
| Moderate | 0.00 | 0.08 | 0.08 |
| Severe | 0.05 | 0.12 | 0.17 |
| Medical History^g^ |  |  |  |
| Cancer | 0.10 | 0.03 | 0.07 |
| Depression^h^ | 0.12 | 0.04 | 0.07 |
| Diabetes | 0.16 | 0.18 | 0.02 |
| Elevated Cholesterol | 0.03 | 0.05 | 0.03 |
| Heart disease | 0.03 | 0.02 | 0.04 |
| Hypertension | 0.10 | 0.01 | 0.11 |
| Respiratory problems | 0.09 | 0.16 | 0.07 |
| Asthma | 0.16 | 0.10 | 0.07 |
| Chronic Obstructive Pulmonary Disease | 0.04 | 0.03 | 0.06 |
| Chronic Bronchitis | 0.02 | 0.00 | 0.01 |
| Emphysema | 0.01 | 0.00 | 0.01 |
| Other^i^ | 0.11 | 0.15 | 0.03 |
| More than one respiratory problem | 0.02 | 0.06 | 0.07 |

^a^ Participants were asked to select “White”, “Black”, or “Other, specify:”. Self-reported “Other” includes: Israeli, Indigenous, Asian, Pilipino, Urdu, Italian, Arab, Trinidadian, Moroccan, Nepalese, Spanish, Tunisian, East Indian.

^b^ Previously used abstinence aids include: Varenicline, Bupropion, Nicotine Patch, Nicotine Gum, Nicotine Inhaler, Nicotine Lozenge, Nicotine QuickMist, Counseling, Other Aids (Acupuncture, Hypnosis, Laser, Apps)

^c^ Motivation to Stop Scale: Possible scores range between 1 and 7, with higher scores indicating stronger motivation to quit smoking. Potential participants completed this 1-item scale during screening and must have selected level 5 or higher to be eligible for the trial, indicating a moderate or strong desire and intention to attempt to quit.

^d^ Fagerström Test for Nicotine Dependence: Possible scores range between 0 and 10, with higher scores indicating a stronger dependence on nicotine. Mild: 0 – 3; Moderate: 4 – 6; Severe: ≥ 7

^e^ Glover-Nilsson Smoking Behavioral Questionnaire: Possible scores range between 0 and 44, with higher scores indicating greater behavioral dependence on smoking. Mild: 0 – 12; Moderate: 12 – 22; Strong: 12 – 33; Very strong: ≥ 34.

^f^ Beck Depression Inventory-II: Possible scores range between 0 and 63, with higher scores indicating greater depressive symptoms. Minimal: 0 – 13; Mild: 14 – 19; Moderate: 20 – 28; Severe: ≥ 29

^g^ Medical history was self-reported

^h^ Defined as prior use of medication for depression

^I^ Other respiratory problems include: chronic pneumonia, shortness of breath, and sleep apnea

**Supplemental Table 2****:** Risk differences (95% CI) for 7-day point prevalence smoking abstinence between treatment groups: primary analysis and sensitivity analyses including self-reported smoking data and multiple imputation

1. Point prevalence abstinence^a^: nicotine e-cigarettes plus counseling vs counseling alone

|  | Primary Analysis – Participants Missing Smoking Data Assumed to Have Returned to Smoking at Baseline Level | | | Sensitivity Analysis – Restricted to Participants with Self-Reported Smoking Data | | | Sensitivity Analysis – Multiple Imputation | | |
| --- | --- | --- | --- | --- | --- | --- | --- | --- | --- |
|  | Abstinence | |  | Abstinence | |  | Abstinence | |  |
| Follow-up | Nicotine  E-Cigarettes  + Counseling | Counseling Alone | Risk Difference  (95% CI) | Nicotine  E-Cigarettes  + Counseling | Counseling Alone | Risk Difference  (95% CI) | Nicotine  E-Cigarettes  + Counseling | Counseling Alone | Risk Difference  (95% CI) |
| Week 1 | 15/128 (11.7%) | 3/121 (2.5%) | 9.2%  (3.0-15.5) | 15/121 (12.4%) | 3/100 (3.0%) | 9.4%  (2.6-16.2) | 16/128 (12.8%) | 5/121 (3.8%) | 9.0%  (1.4-16.6) |
| Week 2 | 25/128 (19.5%) | 6/121 (5.0%) | 14.6%  (6.7-22.5) | 25/120 (20.8%) | 6/89 (6.7%) | 14.1%  (5.2-23.0) | 27/128 (21.1%) | 11/121 (8.8%) | 12.3%  (3.0-21.6) |
| Week 4 | 27/128 (21.1%) | 10/121 (8.3%) | 12.8%  (4.2-21.4) | 27/119 (22.7%) | 10/86 (11.6%) | 11.1%  (0.9-21.2) | 28/128 (22.2%) | 16/121 (12.9%) | 9.3%  (-2.3 to 20.9) |
| Week 8 | 32/128 (25.0%) | 10/121 (8.3%) | 16.7%  (7.8-25.7) | 32/112 (28.6%) | 10/82 (12.2%) | 16.4%  (5.4-27.3) | 35/128 (27.2%) | 20/121 (16.7%) | 10.5%  (-0.7 to 21.7) |
| Week 12 | 28/128 (21.9%) | 11/121 (9.1%) | 12.8%  (4.0-21.6) | 28/115 (24.3%) | 11/83 (13.3%) | 11.1%  (0.4-21.8) | 31/128 (24.1%) | 20/121 (16.5%) | 7.5%  (-5.1 to 20.2) |
| Week 18 | 26/128 (20.3%) | 17/121 (14.0%) | 6.3%  (-3.1 to 15.6) | 26/108 (24.1%) | 17/79 (21.5%) | 2.6%  (-9.6 to 14.7) | 32/128 (24.7%) | 27/121 (22.3%) | 2.4%  (-12.2 to 16.9) |
| Week 24 | 22/128 (17.2%) | 13/121 (10.7%) | 6.4%  (-2.1 to 15.0) | 22/107 (20.6%) | 13/73 (17.8%) | 2.8%  (-8.9 to 14.4) | 28/128 (22.0%) | 21/121 (17.2%) | 4.8%  (-5.5 to 15.2) |
| Week 52 | 30/127 (23.6%) | 12/121 (9.9%) | 13.7%  (4.6-22.8) | 30/103 (29.1%) | 12/69 (17.4%) | 11.7%  (-0.8 to 24.3) | 35/127 (27.9%) | 25/121 (20.5%) | 7.4%  (-5.9 to 20.7) |

Abbreviations: CI = confidence interval.

^a^ Participants were considered abstinent if they abstained from smoking in the 7 days before the visit through a self-report of 0 cigarettes smoked/day, with a carbon monoxide monitor reading ≤ 10 ppm (available for 92%, 87%, 75%, and 76% of self-reported abstinent participants at weeks 4, 12, 24, and 52, respectively)

1. Point prevalence abstinence^a^: nicotine e-cigarettes plus counseling vs non-nicotine e-cigarettes plus counseling

|  | Primary Analysis – Participants Missing Smoking Data Assumed to Have Returned to Smoking at Baseline Level | | | Sensitivity Analysis – Restricted to Participants with Self-Reported Smoking Data | | | Sensitivity Analysis – Multiple Imputation | | |
| --- | --- | --- | --- | --- | --- | --- | --- | --- | --- |
|  | Abstinence | |  | Abstinence | |  | Abstinence | |  |
| Follow-up | Nicotine  E-Cigarettes  + Counseling | Non-Nicotine  E-Cigarettes + Counseling | Risk Difference  (95% CI) | Nicotine  E-Cigarettes  + Counseling | Non-Nicotine  E-Cigarettes  + Counseling | Risk Difference  (95% CI) | Nicotine  E-Cigarettes  + Counseling | Non-Nicotine  E-Cigarettes  + Counseling | Risk Difference  (95% CI) |
| Week 1 | 15/128 (11.7%) | 9/127 (7.1%) | 4.6%  (-2.5 to 11.8) | 15/121 (12.4%) | 9/124 (7.3%) | 5.1%  (-2.3 to 12.6) | 16/128 (12.8%) | 9/127 (7.1%) | 5.7%  (-1.6 to 13.1) |
| Week 2 | 25/128 (19.5%) | 16/127 (12.6%) | 6.9%  (-2.0 to 15.9) | 25/120 (20.8%) | 16/121 (13.2%) | 7.6%  (-1.8 to 17.1) | 27/128 (21.1%) | 17/127 (13.5%) | 7.6%  (-1.9 to 17.0) |
| Week 4 | 27/128 (21.1%) | 16/127 (12.6%) | 8.5%  (-0.6 to 17.6) | 27/119 (22.7%) | 16/121 (13.2%) | 9.5%  (-0.2 to 19.1) | 28/128 (22.2%) | 17/127 (13.4%) | 8.8%  (-1.4 to 19.0) |
| Week 8 | 32/128 (25.0%) | 21/127 (16.5%) | 8.5%  (-1.4 to 18.4) | 32/112 (28.6%) | 21/107 (19.6%) | 9.0%  (-2.3 to 20.2) | 35/128 (27.2%) | 27/127 (20.9%) | 6.2%  (-4.7 to 17.2) |
| Week 12 | 28/128 (21.9%) | 22/127 (17.3%) | 4.6%  (-5.2 to 14.3) | 28/115 (24.3%) | 22/104 (21.2%) | 3.2%  (-7.9 to 14.3) | 31/128 (24.1%) | 26/127 (20.8%) | 3.3%  (-8.3 to 14.9) |
| Week 18 | 26/128 (20.3%) | 24/127 (18.9%) | 1.4%  (-8.3 to 11.2) | 26/108 (24.1%) | 24/101 (23.8%) | 0.3%  (-11.3 to 11.9) | 32/128 (24.7%) | 33/127 (26.3%) | -1.6%  (-13.2 to 9.9) |
| Week 24 | 22/128 (17.2%) | 26/127 (20.5%) | -3.3%  (-12.9 to 6.3) | 22/107 (20.6%) | 26/102 (25.5%) | -4.9%  (-16.3 to 6.5) | 28/128 (22.0%) | 31/127 (24.6%) | -2.5%  (-13.4 to 8.3) |
| Week 52 | 30/127 (23.6%) | 25/127 (19.7%) | 3.9%  (-6.2 to 14.1) | 30/103 (29.1%) | 25/98 (25.5%) | 3.6%  (-8.7 to 15.9) | 35/127 (27.9%) | 30/127 (23.9%) | 3.9%  (-7.7 to 15.6) |

Abbreviations: CI = confidence interval

^a^ Participants were considered abstinent if they abstained from smoking in the 7 days before the visit through a self-report of 0 cigarettes smoked/day, with a carbon monoxide monitor reading ≤ 10 ppm (available for 92%, 87%, 75%, and 76% of self-reported abstinent participants at weeks 4, 12, 24, and 52, respectively)

1. Point prevalence abstinence^a^: non-nicotine e-cigarettes plus counseling vs counseling alone

|  | Primary Analysis – Participants Missing Smoking Data Assumed to Have Returned to Smoking at Baseline Level | | | Sensitivity Analysis – Restricted to Participants with Self-Reported Smoking Data | | | Sensitivity Analysis – Multiple Imputation | | |
| --- | --- | --- | --- | --- | --- | --- | --- | --- | --- |
|  | Abstinence | |  | Abstinence | |  | Abstinence | |  |
| Follow-up | Non-Nicotine  E-Cigarettes  + Counseling | Counseling Alone | Risk Difference  (95% CI) | Non-Nicotine  E-Cigarettes  + Counseling | Counseling Alone | Risk Difference  (95% CI) | Non-Nicotine  E-Cigarettes  + Counseling | Counseling Alone | Risk Difference  (95% CI) |
| Week 1 | 9/127 (7.1%) | 3/121 (2.5%) | 4.6%  (-0.7 to 9.9) | 9/124 (7.3%) | 3/100 (3.0%) | 4.3%  (-1.4% to 9.9) | 9/127 (7.1%) | 5/121 (3.8%) | 3.3%  (-3.0 to 9.6) |
| Week 2 | 16/127 (12.6%) | 6/121 (5.0%) | 7.6%  (0.7-14.6) | 16/121 (13.2%) | 6/89 (6.7%) | 6.5%  (-1.5 to 14.5) | 17/127 (13.5%) | 11/121 (8.8%) | 4.8%  (3.9-13.5) |
| Week 4 | 16/127 (12.6%) | 10/121 (8.3%) | 4.3%  (-3.2 to 11.9) | 16/121 (13.2%) | 10/86 (11.6%) | 1.6%  (-7.5 to 10.7) | 17/127 (13.4%) | 16/121 (12.9%) | 0.5%  (-9.7 to 10.7) |
| Week 8 | 21/127 (16.5%) | 10/121 (8.3%) | 8.3%  (0.2-16.4) | 21/107 (19.6%) | 10/82 (12.2%) | 7.4%  (-2.9 to 17.8) | 27/127 (20.9%) | 20/121 (16.7%) | 4.3%  (-7.6 to 16.2) |
| Week 12 | 22/127 (17.3%) | 11/121 (9.1%) | 8.2%  (-0.1 to 16.6) | 22/104 (21.2%) | 11/83 (13.3%) | 7.9%  (-2.8 to 18.6) | 26/127 (20.8%) | 20/121 (16.5%) | 4.3%  (-9.1 to 17.6) |
| Week 18 | 24/127 (18.9%) | 17/121 (14.0%) | 4.9%  (-4.4 to 14.1) | 24/101 (23.8%) | 17/79 (21.5%) | 2.2%  (-10.1 to 14.5%) | 33/127 (26.3%) | 27/121 (22.3%) | 4.0%  (-9.3 to 17.3) |
| Week 24 | 26/127 (20.5%) | 13/121 (10.7%) | 9.7%  (0.8-18.7) | 26/102 (25.5%) | 13/73 (17.8%) | 7.7%  (-4.5 to 19.9) | 31/127 (24.6%) | 21/121 (17.2%) | 7.4%  (-3.3 to 18.1) |
| Week 52 | 25/127 (19.7%) | 12/121 (9.9%) | 9.8%  (1.0-18.5) | 25/98 (25.5%) | 12/69 (17.4%) | 8.1%  (-4.3 to 20.6) | 30/127 (23.9%) | 25/121 (20.5%) | 3.4%  (-9.6 to 16.5) |

Abbreviations: CI = confidence interval

^a^ Participants were considered abstinent if they abstained from smoking in the 7 days before the visit through a self-report of 0 cigarettes smoked/day, with a carbon monoxide monitor reading ≤ 10 ppm (available for 92%, 87%, 75%, and 76% of self-reported abstinent participants at weeks 4, 12, 24, and 52, respectively)

**Supplemental Table 3****:** Risk differences (95% CI) for continuous smoking abstinence between treatment groups: primary analysis and sensitivity analyses including self-reported smoking data and multiple imputation

1. Continuous abstinence^a^: nicotine e-cigarettes plus counseling vs counseling alone

|  | Primary Analysis – Participants Missing Smoking Data Assumed to Have Returned to Smoking at Baseline Level | | | Sensitivity Analysis – Restricted to Participants with Self-Reported Smoking Data | | | Sensitivity Analysis – Multiple Imputation | | |
| --- | --- | --- | --- | --- | --- | --- | --- | --- | --- |
|  | Abstinence | |  | Abstinence | |  | Abstinence | |  |
| Follow-up | Nicotine  E-Cigarettes + Counseling | Counseling Alone | Risk Difference  (95% CI) | Nicotine  E-Cigarettes  + Counseling | Counseling Alone | Risk Difference  (95% CI) | Nicotine  E-Cigarettes  + Counseling | Counseling Alone | Risk Difference  (95% CI) |
| Week 1 | 15/128 (11.7%) | 3/121 (2.5%) | 9.2%  (3.0-15.5) | 15/121 (12.4%) | 3/100 (3.0%) | 9.4%  (2.6-16.2) | 16/128 (12.8%) | 5/121 (3.8%) | 9.0%  (1.5-16.5) |
| Week 2 | 13/128 (10.2%) | 3/121 (2.5%) | 7.7%  (1.8-13.6) | 13/117 (11.1%) | 3/88 (3.4%) | 7.7%  (0.9-14.5) | 14/128 (10.9%) | 3/121 (2.6%) | 8.3%  (2.0-14.6) |
| Week 4 | 11/128 (8.6%) | 2/121 (1.7%) | 6.9%  (1.6-12.3) | 11/114 (9.6%) | 2/81 (2.5%) | 7.2%  (0.8-13.6) | 11/128 (8.9%) | 3/121 (2.1%) | 6.8%  (0.7-12.8) |
| Week 8 | 9/128 (7.0%) | 1/121 (0.8%) | 6.2%  (1.5-10.9) | 9/110 (8.2%) | 1/74 (1.4%) | 6.8%  (1.1-12.6) | 9/128 (7.0%) | 2/121 (1.3%) | 5.7%  (0.6-10.8) |
| Week 12 | 6/128 (4.7%) | 1/121 (0.8%) | 3.9%  (-0.1 to 7.9) | 6/110 (5.5%) | 1/72 (1.4%) | 4.1%  (-1.0 to 9.1) | 6/128 (4.7%) | 1/121 (1.2%) | 3.5%  (-0.9 to 8.0) |
| Week 18 | 5/128 (3.9%) | 1/121 (0.8%) | 3.1%  (-0.6 to 6.8) | 5/104 (4.8%) | 1/68 (1.5%) | 3.3%  (-1.7 to 8.4) | 5/128 (4.2%) | 1/121 (0.8%) | 3.4%  (-0.6 to 7.3) |
| Week 24 | 5/128 (3.9%) | 1/121 (0.8%) | 3.1%  (-0.6 to 6.8) | 5/101 (5.0%) | 1/63 (1.6%) | 3.4%  (-1.9 to 8.6) | 5/128 (3.9%) | 1/121 (0.8%) | 3.1%  (-0.6 to 6.8) |
| Week 52 | 4/127 (3.1%) | 0/121 (0.0%) | 3.1%  (0.1-6.2) | 4/95 (4.2%) | 0/56 (0.0%) | 4.2%  (0.2-8.3) | 4/127 (3.1%) | 0/121 (0.0%) | 3.2%  (..) |

Abbreviations: CI = confidence interval

^a^ Participants were considered abstinent if they abstained from smoking in the 7 days before the visit through a self-report of 0 cigarettes smoked/day, at all follow-ups since randomization, with a carbon monoxide monitor reading ≤10 ppm (available for 94%, 82%, 78%, and 86% of continuously self-reported abstinent participants at weeks 4, 12, 24, and 52, respectively)

1. Continuous abstinence^a^: nicotine e-cigarettes plus counseling vs non-nicotine e-cigarettes plus counseling

|  | Primary Analysis – Participants Missing Smoking Data Assumed to Have Returned to Smoking at Baseline Level | | | Sensitivity Analysis – Restricted to Participants with Self-Reported Smoking Data | | | Sensitivity Analysis – Multiple Imputation | | |
| --- | --- | --- | --- | --- | --- | --- | --- | --- | --- |
|  | Abstinence | |  | Abstinence | |  | Abstinence | |  |
| Follow-up | Nicotine  E-Cigarettes  + Counseling | Non-Nicotine  E-Cigarettes + Counseling | Risk Difference  (95% CI) | Nicotine  E-Cigarettes  + Counseling | Non-Nicotine  E-Cigarettes  + Counseling | Risk Difference  (95% CI) | Nicotine  E-Cigarettes  + Counseling | Non-Nicotine  E-Cigarettes  + Counseling | Risk Difference  (95% CI) |
| Week 1 | 15/128 (11.7%) | 9/127 (7.1%) | 4.6%  (-2.5 to 11.8) | 15/121 (12.4%) | 9/124 (7.3%) | 5.1%  (-2.3 to 12.6) | 16/128 (12.8%) | 9/127 (7.1%) | 5.7%  (-1.6 to 13.1) |
| Week 2 | 13/128 (10.2%) | 7/127 (5.5%) | 4.6%  (-1.9 to 11.2) | 13/117 (11.1%) | 7/120 (5.8%) | 5.3%  (-1.8 to 12.4) | 14/128 (10.9%) | 7/127 (5.5%) | 5.4%  (-1.4 to 12.2) |
| Week 4 | 11/128 (8.6%) | 5/127 (3.9%) | 4.7%  (-1.3 to 10.6) | 11/114 (9.6%) | 5/118 (4.2%) | 5.4%  (-1.1 to 11.9) | 11/128 (8.9%) | 5/127 (3.9%) | 5.0%  (-1.1 to 11.0) |
| Week 8 | 9/128 (7.0%) | 4/127 (3.1%) | 3.9%  (-1.5 to 9.3) | 9/110 (8.2%) | 4/106 (3.8%) | 4.4%  (-1.9 to 10.7) | 9/128 (7.0%) | 4/127 (3.1%) | 3.9%  (-1.5 to 9.3) |
| Week 12 | 6/128 (4.7%) | 4/127 (3.1%) | 1.5%  (-3.2 to 6.3) | 6/110 (5.5%) | 4/101 (4.0%) | 1.5%  (-4.2 to 7.2) | 6/128 (4.7%) | 4/127 (3.1%) | 1.5%  (-3.2 to 6.3) |
| Week 18 | 5/128 (3.9%) | 3/127 (2.4%) | 1.5%  (-2.7 to 5.8) | 5/104 (4.8%) | 3/95 (3.2%) | 1.7%  (-3.8 to 7.1) | 5/128 (4.2%) | 3/127 (2.4%) | 1.9%  (-2.6 to 6.3) |
| Week 24 | 5/128 (3.9%) | 3/127 (2.4%) | 1.5%  (-2.7 to 5.8) | 5/101 (5.0%) | 3/90 (3.3%) | 1.6%  (-4.0 to 7.2) | 5/128 (3.9%) | 3/127 (2.4%) | 1.5%  (-2.7 to 5.8) |
| Week 52 | 4/127 (3.1%) | 3/127 (2.4%) | 0.8%  (-3.2 to 4.8) | 4/95 (4.2%) | 3/85 (3.5%) | 0.7%  (-5.0 to 6.3) | 4/127 (3.1%) | 3/127 (2.4%) | 0.8%  (-3.2 to 4.8) |

Abbreviations: CI= confidence interval

^a^ Participants were considered abstinent if they abstained from smoking in the 7 days before the visit through a self-report of 0 cigarettes smoked/day, at all follow-ups since randomization, with a carbon monoxide monitor reading ≤ 10 ppm (available for 94%, 82%, 78%, and 86% of continuously self-reported abstinent participants at weeks 4, 12, 24, and 52, respectively)

1. Continuous abstinence^a^: non-nicotine e-cigarettes plus counseling vs counseling alone

|  | Primary Analysis – Participants Missing Smoking Data Assumed to Have Returned to Smoking at Baseline Level | | | Sensitivity Analysis – Restricted to Participants with Self-Reported Smoking Data | | | Sensitivity Analysis – Multiple Imputation | | |
| --- | --- | --- | --- | --- | --- | --- | --- | --- | --- |
|  | Abstinence | |  | Abstinence | |  | Abstinence | |  |
| Follow-up | Non-Nicotine  E-Cigarettes  + Counseling | Counseling Alone | Risk Difference  (95% CI) | Non-Nicotine  E-Cigarettes  + Counseling | Counseling Alone | Risk Difference  (95% CI) | Non-Nicotine  E-Cigarettes  + Counseling | Counseling Alone | Risk Difference  (95% CI) |
| Week 1 | 9/127 (7.1%) | 3/121 (2.5%) | 4.6%  (-0.7 to 9.9) | 9/124 (7.3%) | 3/100 (3.0%) | 4.3%  (-1.4 to 9.9) | 9/127 (7.1%) | 5/121 (3.8%) | 3.3%  (-2.9 to 9.5) |
| Week 2 | 7/127 (5.5%) | 3/121 (2.5%) | 3.0%  (-1.8 to 7.9) | 7/120 (5.8%) | 3/88 (3.4%) | 2.4%  (-3.2 to 8.1) | 7/127 (5.5%) | 3/121 (2.6%) | 2.9%  (-2.1 to 7.8) |
| Week 4 | 5/127 (3.9%) | 2/121 (1.7%) | 2.3%  (-1.8 to 6.4) | 5/118 (4.2%) | 2/81 (2.5%) | 1.8%  (-3.2 to 6.7) | 5/127 (3.9%) | 3/121 (2.1%) | 1.8%  (-2.8 to 6.3) |
| Week 8 | 4/127 (3.1%) | 1/121 (0.8%) | 2.3%  (-1.1 to 5.8) | 4/106 (3.8%) | 1/74 (1.4%) | 2.4%  (-2.1 to 6.9) | 4/127 (3.1%) | 2/121 (1.3%) | 1.8%  (-2.2 to 5.8) |
| Week 12 | 4/127 (3.1%) | 1/121 (0.8%) | 2.3%  (-1.1 to 5.8) | 4/101 (4.0%) | 1/72 (1.4%) | 2.6%  (-2.1 to 7.2) | 4/127 (3.1%) | 1/121 (1.2%) | 2.0%  (-1.9 to 5.9) |
| Week 18 | 3/127 (2.4%) | 1/121 (0.8%) | 1.5%  (-1.6 to 4.6) | 3/95 (3.2%) | 1/68 (1.5%) | 1.7%  (-2.9 to 6.2) | 3/127 (2.4%) | 1/121 (0.8%) | 1.5%  (-1.6 to 4.6) |
| Week 24 | 3/127 (2.4%) | 1/121 (0.8%) | 1.5%  (-1.6 to 4.6) | 3/90 (3.3%) | 1/63 (1.6%) | 1.8%  (-3.1 to 6.6) | 3/127 (2.4%) | 1/121 (0.8%) | 1.5%  (-1.6 to 4.6) |
| Week 52 | 3/127 (2.4%) | 0/121 (0.0%) | 2.4%  (-0.3 to 5.0) | 3/85 (3.5%) | 0/56 (0.0%) | 3.5%  (-0.4 to 7.5) | 3/127 (2.4%) | 0/121 (0.0%) | 2.4%  (..) |

Abbreviations: CI = confidence interval

^a^ Participants were considered abstinent if they abstained from smoking in the 7 days before the visit through a self-report of 0 cigarettes smoked/day, at all follow-ups since randomization, with a carbon monoxide monitor reading ≤ 10 ppm (available for 94%, 82%, 78%, and 86% of continuously self-reported abstinent participants at weeks 4, 12, 24, and 52, respectively)

**Supplemental Table 4****:** Risk differences (95% CI) for mean change in self-reported daily cigarette consumption from baseline between treatment groups: primary analysis and sensitivity analyses including self-reported smoking data and multiple imputation

1. Change in self-reported daily cigarette consumption from baseline^a^: nicotine e-cigarettes plus counseling vs counseling alone

|  | Primary Analysis – Participants Missing Smoking Data Assumed to Have Returned to Smoking at Baseline Level | | | Sensitivity Analysis – Restricted to Participants with Self-Reported Smoking Data | | | Sensitivity Analysis – Multiple Imputation | | | |  |
| --- | --- | --- | --- | --- | --- | --- | --- | --- | --- | --- | --- |
|  | Change in self-reported daily cigarette consumption,  (mean ± SD) | |  | Change in self-reported daily cigarette consumption,  (mean ± SD) | |  | | Change in self-reported daily cigarette consumption,  (mean ± SD) | |  | |
| Follow-up | Nicotine  E-Cigarettes + Counseling | Counseling Alone | Risk Difference  (95% CI) | Nicotine  E-Cigarettes  + Counseling | Counseling Alone | Risk Difference  (95% CI) | | Nicotine  E-Cigarettes  + Counseling | Counseling Alone | Risk Difference  (95% CI) | |
| Week 1 | -13.0 ± 8.3  n=128 | -6.1 ± 9.0  n=121 | -6.9  (-9.1 to -4.7) | -13.1 ± 8.3  n=127 | -6.5 ± 9.1  n=115 | -6.7  (-8.9 to -4.5) | | -13.8 ± 8.0  n=128 | -8.4 ± 9.2  n=121 | -5.4  (-7.6 to -3.1) | |
| Week 2 | -13.9 ± 9.1  n=128 | -6.3 ± 10.3  n=121 | -7.6  (-10.0 to -5.1) | -14.2 ± 8.9  n=125 | -7.0 ± 10.6  n=109 | -7.2  (-9.7 to -4.7) | | -14.6 ± 8.7  n=128 | -9.6 ± 11.0  n=121 | -5.1  (-7.6 to -2.5) | |
| Week 4 | -14.4 ± 8.9  n=128 | -6.6 ± 9.9  n=121 | -7.8  (-10.1 to -5.5) | -14.7 ± 8.7  n=125 | -7.6 ± 10.2  n=105 | -7.1  (-9.6 to -4.7) | | -15.1 ± 8.3  n=128 | -10.9 ± 10.9  n=121 | -4.3  (-6.8 to -1.8) | |
| Week 8 | -13.0 ± 9.8  n=128 | -6.1 ± 9.1  n=121 | -7.3  (-9.7 to -5.0) | -14.3 ± 9.5  n=120 | -7.6 ± 9.6  n=97 | -6.7  (-9.3 to -4.1) | | -14.9 ± 9.5  n=128 | -10.7 ± 10.2  n=121 | -4.2  (-6.7 to -1.6) | |
| Week 12 | -12.6 ± 9.4  n=128 | -7.0 ± 9.3  n=121 | -5.7  (-8.0 to -3.3) | -13.6 ± 9.1  n=119 | -9.2 ± 9.7  n=92 | -4.4  (-7.0 to -1.9) | | -14.1 ± 9.0  n=128 | -11.4 ± 10.3  n=121 | -2.7  (-4.7 to -0.1) | |
| Week 18 | -11.4 ± 10.1  n=128 | -6.9 ± 10.9  n=121 | -4.5  (-7.1 to -1.9) | -12.7 ± 9.9  n=115 | -9.4 ± 11.8  n=89 | -3.3  (-6.3 to -0.3) | | -13.3 ± 9.7  n=128 | -11.5 ± 11.9  n=121 | -1.8  (-4.7 to 1.2) | |
| Week 24 | -10.7 ± 10.1  n=128 | -5.7 ± 9.2  n=121 | -5.0  (-7.4 to -2.5) | -12.0 ± 9.9  n=114 | -7.9 ± 10.0  n=88 | -4.1  (-6.9 to -1.3) | | -12.5 ± 9.8  n=128 | -11.6 ± 12.0  n=121 | -0.9  (-3.8 to 2.0) | |
| Week 52 | -9.5 ± 10.5  n=127 | -5.6 ± 9.5  n=121 | -3.9  (-6.5 to -1.4) | -11.1 ± 10.6  n=109 | -7.9 ± 10.5  n=86 | -3.2  (-6.2 to -0.3) | | -11.4 ± 10.4  n=127 | -11.5 ± 11.6  n=121 | 0.1  (-2.9 to 3.1) | |

Abbreviations: CI = confidence interval; SD= standard deviation

^a^ Mean change in the number of self-reported cigarettes smoked per day in the past week

1. Change in self-reported daily cigarette consumption from baseline^a^: nicotine e-cigarettes plus counseling vs non-nicotine e-cigarettes plus counseling

|  | Primary Analysis – Participants Missing Smoking Data Assumed to Have Returned to Smoking at Baseline Level | | | Sensitivity Analysis – Restricted to Participants with Self-Reported Smoking Data | | | Sensitivity Analysis – Multiple Imputation | | |
| --- | --- | --- | --- | --- | --- | --- | --- | --- | --- |
|  | Change in self-reported daily cigarette consumption,  (mean ± SD) | |  | Change in self-reported daily cigarette consumption,  (mean ± SD) | |  | Change in self-reported daily cigarette consumption,  (mean ± SD) | |  |
| Follow-up | Nicotine  E-Cigarettes  + Counseling | Non-Nicotine  E-Cigarettes + Counseling | Mean Difference  (95% CI) | Nicotine  E-Cigarettes  + Counseling | Non-Nicotine  E-Cigarettes  + Counseling | Risk Difference  (95% CI) | Nicotine  E-Cigarettes  + Counseling | Non-Nicotine  E-Cigarettes  + Counseling | Risk Difference  (95% CI) |
| Week 1 | -13.0 ± 8.3  n=128 | -12.5 ± 10.7  n=127 | -0.6  (-2.9 to 1.8) | -13.1 ± 8.3  n=127 | -12.6 ± 10.7  n=126 | -0.6  (-2.9 to 1.8) | -13.8 ± 8.0  n=128 | -12.9 ± 10.5  n=127 | -0.9  (-3.2 to 1.4) |
| Week 2 | -13.9 ± 9.1  n=128 | -12.4 ± 10.7  n=127 | -1.5  (-4.0 to 0.9) | -14.2 ± 8.9  n=125 | -12.6 ± 10.7  n=125 | -1.7  (-4.1 to 0.8) | -14.6 ± 8.7  n=128 | -13.4 ± 10.7  n=127 | -1.2  (-3.6 to 1.2) |
| Week 4 | -14.4 ± 8.9  n=128 | -11.9 ± 10.0  n=127 | -2.5  (-4.9 to -0.2) | -14.7 ± 8.7  n=125 | -12.2 ± 9.9  n=123 | -2.5  (-4.8 to -0.2) | -15.1 ± 8.3  n=128 | -13.4 ± 10.1  n=127 | -1.7  (-4.1 to 0.6) |
| Week 8 | -13.0 ± 9.8  n=128 | -10.8 ± 11.9  n=127 | -2.6  (-5.3 to 0.1) | -14.3 ± 9.5  n=120 | -11.6 ± 12.0  n=119 | -2.7  (-5.5 to 0.0) | -14.9 ± 9.5  n=128 | -13.9 ± 11.7  n=127 | -1.0  (-3.7 to 1.7) |
| Week 12 | -12.6 ± 9.4  n=128 | -10.6 ± 11.9  n=127 | -2.0  (-4.7 to 0.6) | -13.6 ± 9.1  n=119 | -11.8 ± 12.0  n=114 | -1.8  (-4.5 to 1.0) | -14.1 ± 9.0  n=128 | -13.6 ± 11.7  n=127 | -0.5  (-3.2 to 2.1) |
| Week 18 | -11.4 ± 10.1  n=128 | -9.8 ± 11.9  n=127 | -1.6  (-4.3 to 1.1) | -12.7 ± 9.9  n=115 | -11.0 ± 12.1  n=113 | -1.7  (-4.6 to 1.2) | -13.3 ± 9.7  n=128 | -13.8 ± 11.8  n=127 | 0.4  (-2.3 to 3.2) |
| Week 24 | -10.7 ± 10.1  n=128 | -9.1 ± 11.8  n=127 | -1.6  (-4.3 to 1.1) | -12.0 ± 9.9  n=114 | -10.4 ± 12.1  n=111 | -1.6  (-4.5 to 1.3) | -12.5 ± 9.8  n=128 | -13.0 ± 11.9  n=127 | 0.5  (-2.2 to 3.3) |
| Week 52 | -9.5 ± 10.5  n=127 | -9.3 ± 11.9  n=127 | -0.2  (-3.0 to 2.6) | -11.1 ± 10.6  n=109 | -11.4 ± 12.3  n=104 | 0.3  (-2.8 to 3.4) | -11.4 ± 10.4  n=127 | -12.7 ± 12.1  n=127 | 1.3  (-1.6 to 4.2) |

Abbreviations: CI = confidence interval; SD= standard deviation

^a^ Mean change in the number of self-reported cigarettes smoked per day in the past week

1. Change in self-reported daily cigarette consumption from baseline^a^: non-nicotine e-cigarettes plus counseling vs counseling alone

|  | Primary Analysis – Participants Missing Smoking Data Assumed to Have Returned to Smoking at Baseline Level | | | Sensitivity Analysis – Restricted to Participants with Self-Reported Smoking Data | | | | Sensitivity Analysis – Multiple Imputation | | | | |  |
| --- | --- | --- | --- | --- | --- | --- | --- | --- | --- | --- | --- | --- | --- |
|  | Change in self-reported daily cigarette consumption,  (mean ± SD) | |  | Change in self-reported daily cigarette consumption,  (mean ± SD) | |  | | Change in self-reported daily cigarette consumption,  (mean ± SD) | | |  | |  |
| Follow-up | Non-Nicotine  E-Cigarettes  + Counseling | Counseling Alone | Risk Difference  (95% CI) | Non-Nicotine  E-Cigarettes  + Counseling | Counseling Alone | | Risk Difference  (95% CI) | | Non-Nicotine  E-Cigarettes  + Counseling | Counseling Alone | | Risk Difference  (95% CI) | |
| Week 1 | -12.5 ± 10.7  n=127 | -6.1 ± 9.0  n=121 | -6.4  (-8.8 to -3.9) | -12.6 ± 10.7  n=126 | -6.5 ± 9.1  n=115 | | -6.1  (-8.7 to -3.6) | | -12.9 ± 10.5  n=127 | -8.4 ± 9.2  n=121 | | -4.5  (-7.0 to -2.0) | |
| Week 2 | -12.4 ± 10.7  n=127 | -6.3 ± 10.3  n=121 | -6.0  (-8.6 to -3.4) | -12.6 ± 10.7  n=125 | -7.0 ± 11.6  n=109 | | -5.5  (-8.3 to -2.8) | | -13.4 ± 10.7  n=127 | -9.6 ± 11.0  n=121 | | -3.9  (-6.6 to -1.1) | |
| Week 4 | -11.9 ± 10.0  n=127 | -6.6 ± 9.9  n=121 | -5.3  (-7.8 to -2.8) | -12.2 ± 9.9  n=123 | -7.6 ± 10.2  n=105 | | -4.6  (-7.3 to -2.0) | | -13.4 ± 10.1  n=127 | -10.9 ± 10.9  n=121 | | -2.6  (-5.3 to 0.1) | |
| Week 8 | -10.8 ± 11.9  n=127 | -6.1 ± 9.1  n=121 | -4.8  (-7.4 to -2.1) | -11.6 ± 12.0  n=119 | -7.6 ± 9.6  n=97 | | -4.0  (-6.9 to -1.1) | | -13.9 ± 11.7  n=127 | -10.7 ± 10.2  n=121 | | -3.1  (-6.0 to -0.3) | |
| Week 12 | -10.6 ± 11.9  n=127 | -7.0 ± 9.3  n=121 | -3.6  (-6.3 to -1.0) | -11.8 ± 12.0  n=114 | -9.2 ± 9.7  n=92 | | -2.6  (-5.6 to 0.3) | | -13.6 ± 11.7  n=127 | -11.4 ± 10.3  n=121 | | -2.2  (-5.1 to 0.7) | |
| Week 18 | -9.8 ± 11.9  n=127 | -6.9 ± 10.9  n=121 | -2.9  (-5.8 to -0.0) | -11.0 ± 12.1  n=113 | -9.4 ± 11.8  n=88 | | -1.6  (-5.0 to 1.7) | | -13.8 ± 11.8  n=127 | -11.5 ± 11.9  n=121 | | -2.2  (-5.3 to 0.9) | |
| Week 24 | -9.1 ± 11.8  n=127 | -5.7 ± 9.2  n=121 | -3.4  (-6.0 to -0.7) | -10.4 ± 12.1  n=111 | -7.9 ± 10.0  n=87 | | -2.5  (-5.7 to 0.6) | | -13.0 ± 11.9  n=127 | -11.6 ± 12.0  n=121 | | -1.4  (-4.6 to 1.7) | |
| Week 52 | -9.3 ± 11.9  n=127 | -5.6 ± 9.5  n=121 | -3.7  (-6.4 to -1.0) | -11.4 ± 12.3  n=104 | -7.9 ± 10.5  n=86 | | -3.5  (-6.8 to -0.2) | | -12.7 ± 12.1  n=127 | -11.5 ± 11.6  n=121 | | -1.2  (-4.3 to 1.9) | |

Abbreviations: CI = confidence interval; SD = standard deviation

^a^ Mean change in the number of self-reported cigarettes smoked per day in the past week

**Supplemental Table 5:** Adverse events during the follow-up period (weeks 13-52) of the E3 Trial by treatment group^a^

|  | Nicotine  E-Cigarettes + Individual Counseling | Non-Nicotine  E-Cigarettes + Individual Counseling | Individual Counseling Alone |
| --- | --- | --- | --- |
| **Onset of New Adverse Events**^b^ |  |  |  |
| Participants with new adverse events |  |  |  |
| Cough | 3/27 (11.1%) | 2/28 (7.1%) | 4/41 (9.8%) |
| Dizziness | 5/84 (6.0%) | 6/88 (6.8%) | 4/76 (5.3%) |
| Dry Mouth | 6/46 (13.0%) | 4/45 (8.9%) | 0/58 (0.0%) |
| Dyspnea | 9/60 (15.0%) | 3/50 (6.0%) | 5/64 (7.8%) |
| Headache | 6/48 (12.5%) | 6/46 (13.0%) | 6/63 (9.5%) |
| Indigestion | 9/90 (10.0%) | 8/87 (9.2%) | 3/87 (3.4%) |
| Light Headedness | 6/84 (7.1%) | 6/88 (6.8%) | 4/89 (4.5%) |
| Mouth Irritation | 6/88 (6.8%) | 1/99 (1.0%) | 4/101 (4.0%) |
| Mouth Ulcers | 4/108 (3.7%) | 2/108 (1.9%) | 3/111 (2.7%) |
| Nausea | 8/87 (9.2%) | 6/95 (6.3%) | 6/94 (6.4%) |
| Rhinitis | 15/51 (29.4%) | 6/49 (12.2%) | 5/47 (10.6%) |
| Sore Throat | 9/80 (11.3%) | 8/79 (10.1%) | 4/91 (4.4%) |
| Throat Irritation | 4/53 (7.5%) | 5/67 (7.5%) | 6/84 (7.1%) |
| Vertigo | 4/109 (3.7%) | 7/113 (6.2%) | 4/109 (3.7%) |
| **Reduction of Reported Adverse Events**^c^ |  |  |  |
| Participants no longer experiencing adverse events |  |  |  |
| Cough | 48/101 (47.5%) | 41/99 (41.4%) | 35/80 (43.8%) |
| Dizziness | 27/44 (61.4%) | 25/39 (64.1%) | 37/45 (82.2%) |
| Dry Mouth | 40/82 (48.8%) | 40/82 (48.8%) | 28/63 (44.4%) |
| Dyspnea | 29/68 (42.7%) | 38/77 (49.4%) | 29/57 (50.9%) |
| Headache | 37/80 (46.3%) | 44/81 (54.3%) | 24/58 (41.9%) |
| Indigestion | 21/38 (55.3%) | 24/40 (60.0%) | 22/34 (64.7%) |
| Light Headedness | 26/44 (59.1%) | 26/39 (66.7%) | 16/32 (50.0%) |
| Mouth Irritation | 34/40 (85.0%) | 19/28 (67.9%) | 19/20 (95.0%) |
| Mouth Ulcers | 18/20 (90.0%) | 16/19 (84.2%) | 8/10 (80.0%) |
| Nausea | 32/41 (78.1%) | 23/32 (71.9%) | 15/27 (55.6%) |
| Rhinitis | 37/77 (48.1%) | 38/78 (48.7%) | 45/74 (60.8%) |
| Sore Throat | 32/48 (66.7%) | 34/48 (70.8%) | 25/30 (83.3%) |
| Throat Irritation | 52/75 (69.3%) | 41/60 (68.3%) | 27/37 (73.0%) |
| Vertigo | 15/19 (79.0%) | 10/14 (71.4%) | 8/12 (66.7%) |

^a^ The denominator used to calculate percentages is the total number of participants randomized to each arm. Only the first event for each patient in each category was counted (i.e., the numbers represent the number of patients experiencing an event in each category, rather than the absolute number of events)

^b^ Cumulative sum of potential adverse events reported throughout the follow-up period from week 13 to week 52, obtained by self-report at clinic and telephone visits. Only new adverse events were counted (e.g., the adverse event was not reported at baseline), and only the first report of each potential adverse event was counted for each participant

^c^ Cumulative decrease of reported adverse events from baseline throughout the follow-up period from week 13 to week 52, obtained by self-report at clinic and telephone visits. Only the first reported loss of each potential adverse event was counted for each participant (e.g., the adverse event was reported at baseline, but is no longer reported by the participant)

**Supplemental Table 6:** Evolution of adverse events rates over time from baseline to week 12 compared to adverse events rates over time from baseline to week 52 by treatment group^a^

|  | Nicotine  E-Cigarettes + Individual Counseling | Non-Nicotine  E-Cigarettes + Individual Counseling | Individual Counseling Alone |
| --- | --- | --- | --- |
| **Adverse Events** **from Baseline to Week 12** |  |  |  |
| Cough | 101/128 (78.9%) | 99/127 (78.0%) | 80/121 (66.1%) |
| Dizziness | 44/128 (34.4%) | 39/127 (30.7%) | 45/121 (37.2%) |
| Dry Mouth | 82/128 (64.1%) | 82/127 (64.6%) | 63/121 (52.1%) |
| Dyspnea | 68/128 (53.1%) | 77/127 (60.6%) | 57/121 (47.1%) |
| Headache | 80/128 (62.5%) | 81/127 (63.8%) | 58/121 (47.9%) |
| Indigestion | 38/128 (29.7%) | 40/127 (31.5%) | 34/121 (28.1%) |
| Light Headedness | 44/128 (34.4%) | 39/127 (30.7%) | 32/121 (26.4%) |
| Mouth Irritation | 40/128 (31.3%) | 28/127 (22.0%) | 20/121 (16.5%) |
| Mouth Ulcers | 20/128 (15.6%) | 19/127 (15.0%) | 10/121 (8.3%) |
| Nausea | 41/128 (32.0%) | 32/127 (25.2%) | 27/121 (22.3%) |
| Rhinitis | 77/128 (60.2%) | 78/127 (61.4%) | 74/121 (61.2%) |
| Sore Throat | 48/128 (37.5%) | 48/127 (37.8%) | 30/121 (24.8%) |
| Throat Irritation | 75/128 (58.6%) | 60/127 (47.2%) | 37/121 (30.6%) |
| Vertigo | 19/128 (14.8%) | 14/127 (11.0%) | 12/121 (9.9%) |
|  |  |  |  |
| **Adverse Events** **from Baseline to Week 52** |  |  |  |
| Cough | 104/128 (81.3%) | 101/127 (79.5%) | 84/121 (69.4%) |
| Dizziness | 49/128 (38.3%) | 45/127 (35.4%) | 49/121 (40.5%) |
| Dry Mouth | 88/128 (68.8%) | 86/127 (67.7%) | 63/121 (52.1%) |
| Dyspnea | 77/128 (60.2%) | 80/127 (63.0%) | 62/121 (51.2%) |
| Headache | 86/128 (67.2%) | 87/127 (68.5%) | 64/121 (52.9%) |
| Indigestion | 47/128 (36.7%) | 48/127 (37.8%) | 37/121 (30.6%) |
| Light Headedness | 50/128 (39.1%) | 45/127 (35.4%) | 36/121 (29.8%) |
| Mouth Irritation | 46/128 (35.9%) | 29/127 (22.8%) | 24/121 (19.8%) |
| Mouth Ulcers | 24/128 (18.8%) | 21/127 (16.5%) | 13/121 (10.7%) |
| Nausea | 49/128 (38.3%) | 38/127 (29.9%) | 33/121 (27.3%) |
| Rhinitis | 92/127 (71.9%) | 84/127 (66.1%) | 79/121 (65.3%) |
| Sore Throat | 57/128 (44.5%) | 56/127 (44.1%) | 34/121 (28.1%) |
| Throat Irritation | 79/128 (61.7%) | 65/127 (51.2%) | 43/121 (35.5%) |
| Vertigo | 23/128 (18.0%) | 21/127 (16.5%) | 16/121 (13.2%) |

^a^ The denominator used to calculate percentages is the total number of participants randomized to each arm. Only the first event for each patient in each category was counted (i.e., the numbers represent the number of patients experiencing an event in each category, rather than the absolute number of events)

**Supplemental Table 7:** Serious adverse events occurring between 24 and 52 weeks follow-up by treatment group

| **Serious Adverse Event** | **Age^a^** | **Sex** | **Previous Medical History** | **Years Smoked^a^** | | **Cigarettes Per Day^a^** | **Time to Event (Days)** | **E-Cigarette Discont to Event (Days)^b^** | **Death** | **Use of**  **Non-Study**  **E-Cigarette^c^** |
| --- | --- | --- | --- | --- | --- | --- | --- | --- | --- | --- |
| **Nicotine E-Cigarettes + Counseling** | | | | | | | | |  |  |
| COPD Exacerbation, Esophageal Candidiasis | 59 | M | Emphysema, High Cholesterol, Gangrene Right Foot, Right Leg Amputation, PVD, Restless Leg Syndrome, Spondylosis Cervical | 49 | | 37 | 292 | 218 | No | Yes^d^ |
| Critical left limb ischemia, PVD |  |  |  |  |  |  | 306 | 232 | No | No |
| Colitis NYD | 60 | F | Asthma, Chronic bronchitis, Constipation, Depression, Edema, GERD, Insomnia | 48 | | 10 | 210 | 120 | No | No |
| Heart Attack Due to Hypertension | 42 | M | Anxiety, Depression, Diabetes, High cholesterol, Hypertension | 28 | | 40 | 225 | 141 | Yes | Yes^e^ |
| Miscarriage | 34 | F | None | 16 | | 10 | 224 | 176 | No | No |
| Septic shock for MSSA bacteremia | 57 | M | Angina, Bypass, Arthritis, Depression, Degenerative Disc Disease, Diabetes, Heart attack, High Cholesterol, Hypertension, Stroke | 41 | | 50 | 241 | 0^f^ | No | No |
| Progressive SOB |  |  |  |  |  |  | 270 | 29 | No |  |
| Mobility problem secondary to CHS and AKI |  |  |  |  |  |  | 312 | 71 | No |  |
| Stroke | 62 | M | Anemia, Insomnia, High Cholesterol, Pernicious | 46 | | 15 | 197 | 197 | No | No |
| Transient Ischemic Attack | 49 | M | Angioplasty, Heart attack, High Cholesterol, Hypertension | 36 | | 25 | 341 | 257 | No | Yes^g^ |
| **Non-Nicotine E-Cigarettes + Counseling** | | | | | | | | |  |  |
| Critical Lower Limb Ischemia | 66 | M | Angioplasty, Diabetes, Myocardial Infarction, High Cholesterol, Hypertension | 50 | |  | 323 | 235 | No | No |
| Accidental Overdose of Clonazepam and Ativan | 58 | M | Asthma, COPD, HIV Positive, Hypertension, GERD, Insomnia, Psoriasis, Psoriatic Arthritis | 44 | | 25 | 209 | 132 | No | No |
| Seizure |  |  |  |  |  |  | 308 | 231 | No |  |
| COPD Exacerbation | 54 | F | Anxiety, Asthma, COPD, Depression, High Cholesterol, Hypertension, Hypothyroidism, GERD, Osteoarthritis | 43 | | 25 | 241 | 64 | No | No |
| Pulmonary Carcinoma | 68 | F | Angina, COPD, Chronic Pneunomia, Depression, Diabetes, High Cholesterol, Hypertension | 55 | | 37 | 184 | 107 | No | No |
| Stroke | 61 | M | Chronic Bronchitis, COPD, High Cholesterol, Skin Cancer (1 year remission), Sleep Apnea | 49 | | 28 | 252 | 169 | No | No |
| Magnesium concentration blood imbalance | 71 | M | Diabetes, Heart Attack, Hypertension | 52 | | 23 | 320 | 284 | No | No |
| **Counseling Alone** | | | | | | | | |  |  |
| Right Above-Knee Amputation | 48 | M | Diabetes, Abdominal Tumour & Neuroblastoma (47 years remission), High cholesterol | 35 | | 25 | 288 | N/A | No | No |
| Urinary Tract Infection |  |  |  |  |  |  | 315 | N/A | No |  |
| Acute Pelvic Inflammatory Disease | 26 | F | Anxiety, Depression, Insomnia | | 13 | 25 | 217 | N/A | No | No |
| Miscarriage | 45 | F | None | | 25 | 12 | 205 | N/A | No | No |
| Multiple Sclerosis | 49 | F | Depression, High Cholesterol, Occasional SOB | | 37 | 25 | 337 | N/A | No | No |
| Stroke | 53 | M | None | 36 | | 25 | 334 | N/A | No | No |

Abbreviations: AKI = acute kidney injury; CHS = cannabinoid hyperemesis syndrome; COPD = chronic obstructive pulmonary disease; Discont = discontinuation; GERD = gastroesophageal reflux disease; HIV = human immunodeficiency virus; MSSA = methicillin-susceptible staphylococcus aureus; N/A = not applicable; NYD = not yet diagnosed; PVD = peripheral vascular disease; SOB = shortness of breath

^a^ At time of randomization into the E3 Trial

^b^ A value of 0 indicates patient was using the study-provided e-cigarette at time of event

^c^ “No” indicates that the participant was not using a non-study e-cigarette at the time of the event

^d^ Participant was using a non-study non-nicotine e-cigarette at the time of their event. The use of non-study e-cigarette was initiated after the week 12 treatment period

^e^ Participant was still using the study provided nicotine e-cigarette at the time of the event. The participant did not return the study e-cigarette at the end of the 12-week treatment period and kept using it (150 days post treatment period)

^f^ Participant was possibly using a non-study nicotine e-cigarette at the time of their event. The use of non-study e-cigarette was initiated after the week 12 treatment period

^g^ Participant initiated a non-study nicotine e-cigarette after the end of the treatment but was not using the non-study e-cigarette at the time of the event

**Supplemental Table 8:** Risk differences (95% CI) for week 52 7-day point prevalence smoking abstinence^a^ between treatment groups abstinence by baseline characteristics.

|  |  |  |  | Risk Differences (95% CI) | | |
| --- | --- | --- | --- | --- | --- | --- |
| Characteristic | Nicotine  E-Cigarettes  + Individual Counseling | Non-Nicotine  E-Cigarettes  + Individual Counseling | Individual Counseling  Alone | Nicotine  E-Cigarettes  + Individual Counseling  vs  Individual Counseling Alone | Nicotine  E-Cigarettes  + Individual Counseling  vs  Non-Nicotine  E-Cigarettes  + Individual Counseling | Non-Nicotine  E-Cigarettes  + Individual Counseling  vs  Individual Counseling Alone |
| Demographic Characteristics |  |  |  |  |  |  |
| Sex |  |  |  |  |  |  |
| Male | 14/62 (22.6%) | 16/71 (22.5%) | 7/64 (10.9%) | 11.6%  (-1.3 to 24.6) | 0.1%  (-14.2 to 14.3) | 11.6%  (-0.8 to 24.0) |
| Female | 16/65 (24.6%) | 9/56 (16.1%) | 5/57 (8.8%) | 15.8%  (3.1-28.6) | 8.5%  (-5.7 to 22.8) | 7.3%  (-4.8 to 19.4) |
| Age |  |  |  |  |  |  |
| Under 40 | 10/25 (40.0%) | 8/26 (30.8%) | 3/18 (16.7%) | 23.3%  (-2.5 to 49.1) | 9.2%  (-16.9 to 35.4) | 14.1%  (-10.6 to 38.8) |
| 40 to 50 | 6/17 (35.3%) | 2/20 (10.0%) | 2/27 (7.4%) | 27.9%  (3.1-52.7) | 25.3%  (-1.0 to 51.5) | 2.6%  (-13.9 to 19.0) |
| 50 to 60 | 6/44 (13.6%) | 7/46 (15.2%) | 3/43 (7.0%) | 6.7%  (-6.0 to 19.3) | -1.6%  (-16.1 to 12.9) | 8.2%  (-4.6 to 21.1) |
| 60 and over | 8/41 (19.5%) | 8/35 (22.9%) | 4/33 (12.1%) | 7.4%  (-9.1 to 23.9) | -3.3%  (-21.8 to 15.1) | 10.7%  (-7.1% to 28.6) |
| Self-Reported Race |  |  |  |  |  |  |
| White | 27/120 (22.5%) | 21/111 (18.9%) | 10/104 (9.6%) | 12.9%  (3.5-22.3) | 3.6%  (-6.9 to 14.0) | 9.3%  (0.1-18.5) |
| Black | 1/1 (100.0%) | 3/7 (42.9%) | 0/3 (0.0%) | 100.0%  (100.0-100.0) | 57.1%  (20.5-93.8) | 42.9%  (6.2-79.5) |
| Other^b^ | 2/6 (33.3%) | 1/9 (11.1%) | 2/14 (14.3%) | 19.1%  (-22.9 to 61.0) | 22.2%  (-20.7 to 65.2) | -3.2%  (-30.7 to 24.4) |
| Education |  |  |  |  |  |  |
| Completed high school or less | 6/47 (12.8%) | 10/48 (20.8%) | 2/47 (4.3%) | 8.5%  (-2.6 to 19.7) | -8.1%  (-23.0 to 6.9) | 16.6%  (3.7-29.4) |
| More than high school | 24/80 (30.0%) | 15/79 (19.0%) | 10/74 (13.5%) | 16.5%  (3.8-29.2) | 11.0%  (-2.2 to 24.3) | 5.5%  (-6.2 to 17.1) |
| Smoking Characteristics |  |  |  |  |  |  |
| Years Smoked |  |  |  |  |  |  |
| Under 20 | 8/24 (33.3%) | 7/24 (29.2%) | 1/21 (4.8%) | 28.6%  (7.6-49.5) | 4.2%  (-22.0 to 30.4) | 24.4%  (4.1-44.7) |
| 20 to 35 | 10/28 (35.7%) | 4/25 (16.0%) | 5/31 (16.1%) | 19.6%  (-2.4 to 41.6) | 19.7%  (-3.1 to 42.6) | -0.1%  (-19.5 to 19.2) |
| 35 to 50 | 10/61 (16.4%) | 12/63 (19.0%) | 4/59 (6.8%) | 9.6%  (-1.7 to 20.9) | -2.7%  (-16.1 to 10.8) | 12.3%  (0.6-23.9) |
| 50 and over | 2/14 (14.3%) | 2/15 (13.3%) | 2/10 (20.0%) | -5.7%  (-36.6 to 25.1) | 1.0%  (-24.2 to 26.1) | -6.7%  (-36.8 to 23.5) |
| Average cigarettes/day |  |  |  |  |  |  |
| Under 20 | 19/59 (32.2%) | 15/54 (27.8%) | 10/55 (18.2%) | 14.0%  (-1.7 to 29.7) | 4.4%  (-12.5 to 21.3) | 9.6%  (-6.1 to 25.3) |
| 20 and over | 11/68 (16.2%) | 10/73 (13.7%) | 2/66 (3.0%) | 13.2%  (3.5-22.8) | 2.5%  (-9.3 to 14.3) | 10.7%  (1.8-19.6) |
| Previous number of attempts to quit | |  |  |  |  |  |
| Never | 4/12 (33.3%) | 2/9 (22.2%) | 1/13 (7.7%) | 25.6%  (-4.7 to 56.0) | 11.1%  (-27.0 to 49.2) | 14.5%  (-16.3 to 45.3) |
| 1 time | 8/26 (30.8%) | 6/28 (21.4%) | 3/24 (12.5%) | 18.3%  (-3.9 to 40.4) | 9.3%  (-14.0 to 32.7) | 8.9%  (-11.2 to 29.1) |
| 2 times | 0/26 (0.0%) | 5/31 (16.1%) | 3/19 (15.8%) | -15.8%  (-32.2 to 0.6) | -16.1%  (-29.1 to -3.2) | 0.3%  (-20.6 to 21.2) |
| 3 times and more | 18/63 (28.6%) | 12/59 (20.3%) | 5/65 (7.7%) | 20.9%  (8.0-33.8) | 8.2%  (-6.9 to 23.4) | 12.7%  (0.5-24.8) |
| Previous use of abstinence aids^c^ | |  |  |  |  |  |
| No | 6/26 (23.1%) | 5/27 (18.5%) | 3/22 (13.6%) | 9.4%  (-12.2 to 31.1) | 4.6%  (-17.3 to 26.4) | 4.9%  (-15.6 to 25.4) |
| Yes | 24/101 (23.8%) | 20/100 (20.0%) | 9/99 (9.1%) | 14.7%  (4.6-24.7) | 3.8%  (-7.7 to 15.2) | 10.9%  (1.2-20.6) |
| Previous use of an e-cigarette |  |  |  |  |  |  |
| No | 14/73 (19.2%) | 14/79 (17.7%) | 9/88 (10.2%) | 9.0%  (-2.1 to 20.0) | 1.5%  (-10.9 to 13.8) | 7.5%  (-3.0 to 18.0) |
| Yes | 16/54 (29.6%) | 11/48 (22.9%) | 3/33 (9.1%) | 20.5%  (4.9-36.2) | 6.7%  (-10.3 to 23.7) | 13.8%  (-1.6 to 29.2) |
| Other smoker(s) at home |  |  |  |  |  |  |
| No | 22/88 (25.0%) | 18/82 (22.0%) | 11/85 (12.9%) | 12.1%  (0.5-23.6) | 3.1%  (-9.7 to 15.8) | 9.0%  (-2.4 to 20.5) |
| Yes | 8/39 (20.5%) | 7/45 (15.6%) | 1/36 (2.8%) | 17.7%  (4.0-31.5) | 5.0%  (-11.6 to 21.5) | 12.8%  (0.9-24.7) |
| Other Lifestyle Characteristics |  |  |  |  |  |  |
| Body Mass Index > 30 kg/m^2^ |  |  |  |  |  |  |
| No | 20/80 (25.0%) | 12/80 (15.0%) | 8/75 (10.7%) | 14.3%  (2.6-26.1) | 10.0%  (-2.3 to 22.3) | 4.3%  (-6.2 to 14.8) |
| Yes | 10/47 (21.3%) | 13/47 (27.7%) | 4/45 (8.9%) | 12.4%  (-2.0 to 26.7) | -6.4%  (-23.7 to 11.0) | 18.8%  (3.5-34.0) |
| *Missing* | *0* | *0* | *1* |  |  |  |
| Alcoholic drinks/week |  |  |  |  |  |  |
| None | 6/42 (14.3%) | 9/55 (16.4%) | 1/35 (2.9%) | 11.4%  (-0.5 to 23.4) | -2.1%  (-16.5 to 12.3) | 13.5%  (2.3-24.7) |
| 1 drink/week | 6/32 (18.8%) | 7/20 (35.0%) | 4/28 (14.3%) | 4.5%  (-14.3 to 23.2) | -16.3%  (-41.2 to 8.7) | 20.7%  (-3.9 to 45.3) |
| 3 drinks/week | 3/14 (21.4%) | 2/16 (12.5%) | 2/20 (10.0%) | 11.4%  (-13.8 to 36.6) | 8.9%  (-18.0 to 35.9) | 2.5%  (-18.4 to 23.4) |
| More than 3 drinks/week | 15/39 (38.5%) | 7/36 (19.4%) | 5/37 (13.5%) | 25.0%  (6.1-43.8) | 19.0%  (-1.0 to 39.0) | 5.9%  (-11.1 to 22.9) |
| *Missing* | *0* | *0* | *2* |  |  |  |
| Questionnaires |  |  |  |  |  |  |
| Fagerström Test for Nicotine Dependence^d^ | |  |  |  |  |  |
| Mild | 3/19 (15.8%) | 9/25 (36.0%) | 1/21 (4.8%) | 11.0  (-7.7 to 29.8) | -20.2%  (-45.2 to 4.8) | 31.2%  (10.3-52.1) |
| Moderate | 18/59 (30.5%) | 11/57 (19.3%) | 9/54 (16.7%) | 13.8%  (-1.6 to 29.2) | 11.2%  (-4.4. to 26.8) | 2.6%  (-11.6 to 16.9) |
| Severe | 9/49 (18.4%) | 5/45 (11.1%) | 2/44 (4.6%) | 13.8%  (1.4-26.3) | 7.3%  (-7.0 to 21.5) | 6.6%  (-4.5 to 17.6) |
| *Missing* | *0* | *0* | *2* |  |  |  |
| Glover-Nilsson Smoking Behavioral Questionnaire^e^ | | |  |  |  |  |
| Mild | 2/17 (11.8%) | 6/25 (24.0%) | 2/22 (9.1%) | 2.7%  (-16.8 to 22.1) | -12.2%  (-34.9 to 10.5) | 14.9%  (-5.7 to 35.5) |
| Moderate | 17/59 (28.8%) | 11/55 (20.0%) | 7/53 (13.2%) | 15.6%  (0.9-30.3) | 8.8%  (-6.9 to 24.5) | 6.8%  (-7.2 to 20.8) |
| Strong | 10/42 (23.8%) | 6/36 (16.7%) | 3/33 (9.1%) | 14.7%  (-1.5 to 30.9) | 7.1%  (-10.6 to 24.9) | 7.6%  (-8.1 to 23.2) |
| Very Strong | 1/9 (11.1%) | 2/10 (20.0%) | 0/11 (0.0%) | 11.1%  (-9.4 to 31.6) | -8.9%  (-41.1 to 23.3) | 20.0%  (-4.8 to 44.8) |
| *Missing* | *0* | *1* | *2* |  |  |  |
| Beck Depression Inventory-II^f^ |  |  |  |  |  |  |
| Minimal | 24/86 (27.9%) | 18/92 (19.6%) | 8/78 (10.3%) | 17.7%  (6.0-29.3) | 8.3%  (-4.1 to 20.8) | 9.3%  (-1.2 to 19.9) |
| Mild | 2/19 (10.5%) | 3/19 (15.8%) | 2/18 (11.1%) | -0.6%  (-20.6 to 19.5) | -5.3%  (-26.7 to 16.2) | 4.7%  (-17.2 to 26.6) |
| Moderate | 2/14 (14.3%) | 3/12 (25.0%) | 0/14 (0.0%) | 14.3%  (-4.0 to 32.6) | -10.7%  (-41.3 to 19.9) | 25.0%  (0.5-49.5) |
| Severe | 1/7 (14.3%) | 1/4 (25.0%) | 1/8 (12.5%) | 1.8%  (-32.8 to 36.4) | -10.7%  (-60.4 to 39.0) | 12.5%  (-35.7 to 60.7) |
| *Missing* | *1* | *0* | *3* |  |  |  |
| Medical History^g^ |  |  |  |  |  |  |
| Cancer |  |  |  |  |  |  |
| No | 29/116 (25.0%) | 23/115 (20.0%) | 10/107 (9.3%) | 15.7%  (6.0-25.3) | 5.0%  (-5.8 to 15.8) | 10.7%  (1.5-19.8) |
| Yes | 1/11 (9.1%) | 2/12 (16.7%) | 2/14 (14.3%) | -5.2%  (-30.2 to 19.8) | -7.6%  (-34.7 to 19.5) | 2.4%  (-25.6 to 30.3) |
| Depression^h^ |  |  |  |  |  |  |
| No | 22/83 (26.5%) | 20/85 (23.5%) | 8/85 (9.4%) | 17.1%  (5.8-28.4) | 3.0%  (-10.1 to 16.1) | 14.1%  (3.2-25.1) |
| Yes | 8/44 (18.2%) | 5/42 (11.9%) | 4/36 (11.1%) | 7.1%  (-8.3 to 22.4) | 6.3%  (-8.8 to 21.3) | 0.8%  (-13.4 to 15.0) |
| Diabetes |  |  |  |  |  |  |
| No | 27/112 (24.1%) | 20/103 (19.4%) | 12/99 (12.1%) | 12.0%  (1.8-22.2) | 4.7%  (-6.3 to 15.7) | 7.3%  (-2.7 to 17.3) |
| Yes | 3/15 (20.0%) | 5/24 (20.8%) | 0/22 (0.0%) | 20.0%  (-0.2 to 40.2) | -0.8%  (-26.8 to 25.1) | 20.8%  (4.6-37.1) |
| Elevated Cholesterol |  |  |  |  |  |  |
| No | 21/81 (25.9%) | 14/77 (18.2%) | 9/75 (12.0%) | 13.9%  (1.9-26.0) | 7.7%  (-5.1 to 20.6) | 6.2%  (-5.2 to 17.5) |
| Yes | 9/46 (19.6%) | 11/50 (22.0%) | 3/46 (6.5%) | 13.0%  (-0.5 to 26.6) | -2.4%  (-18.7 to 13.8) | 15.5%  (2.0-29.0) |
| Heart disease |  |  |  |  |  |  |
| No | 24/104 (23.1%) | 22/105 (21.0%) | 10/98 (10.2%) | 12.9%  (2.8-23.0) | 2.1%  (-9.1 to 13.4) | 10.8%  (0.9-20.6) |
| Yes | 6/23 (26.1%) | 3/22 (13.6%) | 2/23 (8.7%) | 17.4%  (-3.9 to 38.7) | 12.5%  (-10.5 to 35.4) | 4.9%  (-13.5 to 23.3) |
| Hypertension |  |  |  |  |  |  |
| No | 21/87 (24.1%) | 17/86 (19.8%) | 11/88 (12.5%) | 11.6%  (0.3-23.0) | 4.4%  (-8.0 to 16.7) | 7.3%  (-3.6 to 18.2) |
| Yes | 9/40 (22.5%) | 8/41 (19.5%) | 1/33 (3.0%) | 19.5%  (5.3-33.7) | 3.0%  (-14.8 to 20.7) | 16.5%  (3.0-30.0) |
| Respiratory problems |  |  |  |  |  |  |
| No | 28/96 (29.2%) | 20/87 (23.0%) | 11/87 (12.6%) | 16.5%  (5.1-28.0) | 6.2%  (-6.5 to 18.9) | 10.3%  (-0.9 to 21.6) |
| Yes | 2/31 (6.5%) | 5/40 (12.5%) | 1/34 (2.9%) | 3.5%  (-6.8 to 13.9) | -6.1%  (-19.5 to 7.4) | 9.6%  (-2.2 to 21.3) |

Abbreviations: CI = confidence interval

^a^ Includes all participants except those who died. Participants were analyzed according to the group to which they were randomized. Participants who were lost-to-follow-up or withdrew were assumed to have returned to smoking at their baseline level

^b^ Participants were asked to select “White”, “Black”, or “Other, specify:”. Self-reported “Other” includes: Israeli, Indigenous, Asian, Pilipino, Urdu, Italian, Arab, Trinidadian, Moroccan, Nepalese, Spanish, Tunisian, East Indian

^c^ Previously used abstinence aids include: Varenicline, Bupropion, Nicotine Patch, Nicotine Gum, Nicotine Inhaler, Nicotine Lozenge, Nicotine QuickMist, Counseling, Other Aids (Acupuncture, Hypnosis, Laser, Apps)

^d^ Fagerström Test for Nicotine Dependence: Possible scores range between 0 and 10, with higher scores indicating a stronger dependence on nicotine. Mild: 0 – 3; Moderate: 4 – 6; Severe: ≥ 7

^e^ Glover-Nilsson Smoking Behavioral Questionnaire: Possible scores range between 0 and 44, with higher scores indicating greater behavioral dependence on smoking. Mild: 0 – 12; Moderate: 12 – 22; Strong: 12 – 33; Very strong: ≥ 34

^f^ Beck Depression Inventory-II: Possible scores range between 0 and 63, with higher scores indicating greater depressive symptoms. Minimal: 0 – 13; Mild: 14 – 19; Moderate: 20 – 28; Severe: ≥ 29

^g^ Medical history was self-reported

^h^ Defined as prior use of medication for depression

**Supplemental Table 9:** Description of baseline characteristics of participants by self-reported smoking data availability at 52 weeks*

| Characteristic | All Participants  (n = 375) | Smoking Data Available  (n = 270) | Smoking Data Missing  (n = 105) | Standardized Differences (Available vs Missing) |
| --- | --- | --- | --- | --- |
| Demographic Characteristics |  |  |  |  |
| Age, mean years ± SD | 51.8 ± 12.5 | 51.3 ± 12.5 | 53.1 ± 12.6 | 0.15 |
| Sex |  |  |  |  |
| Male | 197 (52.5%) | 143 (53.0%) | 54 (51.4%) | 0.03 |
| Female | 178 (47.5%) | 127 (47.0%) | 51 (48.6%) | 0.03 |
| Self-Reported Race |  |  |  |  |
| White | 335 (89.3%) | 237 (87.8%) | 98 (93.3%) | 0.19 |
| Black | 11 (2.9%) | 8 (3.0%) | 3 (2.9%) | 0.01 |
| Other^a^ | 29 (7.7%) | 25 (9.3%) | 4 (3.8%) | 0.22 |
| Education |  |  |  |  |
| More than high school | 233 (62.1%) | 175 (64.8%) | 58 (55.2%) | 0.20 |
| Smoking Characteristics |  |  |  |  |
| Years smoked, mean ± SD | 34.2 ± 13.7 | 33.5 ± 13.8 | 36.2 ± 13.5 | 0.20 |
| Cigarettes/day at baseline, mean ± SD | 21.1 ± 10.6 | 20.5 ± 10.5 | 22.5 ± 10.5 | 0.19 |
| Previously attempted to quit | 341 (90.9%) | 239 (88.5%) | 102 (97.1%) | 0.34 |
| Median number of serious attempts to quit (IQR) | 3 (2 – 5) | 3 (2 – 5) | 3 (2 – 4) | NR |
| Previously used abstinence aids^b^ for smoking cessation | 300 (80.0%) | 215 (79.6%) | 85 (81.0%) | 0.03 |
| Previously tried an e-cigarette | 135 (36.0%) | 101 (37.4%) | 34 (32.4%) | 0.11 |
| Other smoker(s) at home | 120 (32.0%) | 83 (30.7%) | 37 (35.2%) | 0.10 |
| Other Lifestyle Characteristics |  |  |  |  |
| Body Mass Index ≥ 30 kg/m^2^ | 139 (37.1%) | 103 (38.1%) | 36 (34.3%) | 0.08 |
| Alcoholic drinks/week, mean ± SD | 3.6 ± 6.7 | 3.6 ± 6.9 | 3.5 ± 6.2 | 0.02 |
| Questionnaires | | | | |
| Motivation to Stop Scale^c^ | n = 375 | n = 270 | n = 105 |  |
| Mean Score ± SD | 6.1 ± 0.8 | 6.1 ± 0.8 | 6.2 ± 0.9 | 0.11 |
| 5 (‘I want to stop smoking and hope to soon’) | 109 (29.1%) | 79 (29.2%) | 30 (28.6%) | 0.02 |
| 6 (‘I really want to stop smoking and intend to in the next 3 months’) | 111 (29.6%) | 86 (31.9%) | 25 (23.8%) | 0.18 |
| 7 (‘I really want to stop smoking and intend to in the next month’) | 155 (41.3%) | 105 (38.9%) | 50 (47.6%) | 0.18 |
| Fagerström Test for Nicotine Dependence^d^ | n = 374 | n = 269 | n = 104 |  |
| Mean Score ± SD | 5.7 ± 2.2 | 5.6 ± 2.2 | 6.0 ± 2.2 | 0.16 |
| Mild | 65 (17.3%) | 50 (18.5%) | 15 (14.3%) | 0.11 |
| Moderate | 170 (45.3%) | 122 (45.2%) | 48 (45.7%) | 0.02 |
| Severe | 138 (36.8%) | 97 (35.9%) | 41 (39.0%) | 0.07 |
| Glover Nilsson Smoking Behavioral Questionnaire^e^ | n = 373 | n = 267 | n = 105 |  |
| Mean Score ± SD | 20.5 ± 8.2 | 20.6 ± 8.3 | 20.1 ± 8.1 | 0.06 |
| Mild | 64 (17.1%) | 45 (16.7%) | 19 (18.1%) | 0.03 |
| Moderate | 167 (44.5%) | 117 (43.3%) | 50 (47.6%) | 0.08 |
| Strong | 111 (29.6%) | 84 (31.1%) | 27 (25.7%) | 0.13 |
| Very Strong | 30 (8.0%) | 21 (7.8%) | 9 (8.6%) | 0.03 |
| Beck Depression Inventory-II^f^ | n = 372 | n = 267 | n = 104 |  |
| Mean Score ± SD | 10.7 ± 9.2 | 11.3 ± 9.6 | 8.9 ± 8.1 | 0.27 |
| Minimal | 257 (68.5%) | 178 (65.9%) | 78 (74.3%) | 0.18 |
| Mild | 56 (14.9%) | 42 (15.6%) | 14 (13.3%) | 0.06 |
| Moderate | 40 (10.7%) | 30 (11.1%) | 10 (9.5%) | 0.05 |
| Severe | 19 (5.1%) | 17 (6.3%) | 2 (1.9%) | 0.22 |
| Medical History^g^ |  |  |  |  |
| Cancer | 37 (9.9%) | 25 (9.3%) | 12 (11.4%) | 0.07 |
| Depression^h^ | 122 (32.5%) | 87 (32.2%) | 35 (33.3%) | 0.02 |
| Diabetes | 61 (16.3%) | 41 (15.2%) | 20 (19.0%) | 0.10 |
| Elevated Cholesterol | 142 (37.9%) | 103 (38.1%) | 39 (37.1%) | 0.02 |
| Heart disease | 68 (18.1%) | 40 (14.8%) | 28 (26.7%) | 0.30 |
| Hypertension | 114 (30.4%) | 83 (30.7%) | 31 (29.5%) | 0.03 |
| Respiratory problems | 105 (28.0%) | 71 (26.3%) | 34 (32.4%) | 0.13 |
| Asthma | 52 (13.9%) | 34 (12.6%) | 18 (17.1%) | 0.13 |
| Chronic Obstructive Pulmonary Disease | 38 (10.1%) | 25 (9.3%) | 13 (12.4%) | 0.10 |
| Chronic Bronchitis | 30 (8.0%) | 22 (8.1%) | 8 (7.6%) | 0.02 |
| Emphysema | 15 (4.0%) | 10 (3.7%) | 5 (4.8%) | 0.05 |
| Other^i^ | 11 (2.9%) | 6 (2.2%) | 5 (4.8%) | 0.14 |
| More than one respiratory problem | 31 (8.3%) | 22 (8.1%) | 9 (8.6%) | 0.02 |

Abbreviations: NR = not relevant; SD = standard deviation

*Data presented as count (%), mean ± SD, or median (Q1-Q3)

^a^ Participants were asked to select “White”, “Black”, or “Other, specify:”. Self-reported “Other” includes: Israeli, Indigenous, Asian, Pilipino, Urdu, Italian, Arab, Trinidadian, Moroccan, Nepalese, Spanish, Tunisian, East Indian

^b^ Previously used abstinence aids include: Varenicline, Bupropion, Nicotine Patch, Nicotine Gum, Nicotine Inhaler, Nicotine Lozenge, Nicotine QuickMist, Counseling, Other Aids (Acupuncture, Hypnosis, Laser, Apps)

^c^ Motivation to Stop Scale: Possible scores range between 1 and 7, with higher scores indicating stronger motivation to quit smoking. Potential participants completed this 1-item scale during screening, and must have selected level 5 or higher to be eligible for the trial, indicating a moderate or strong desire and intention to attempt to quit

^d^ Fagerström Test for Nicotine Dependence: Possible scores range between 0 and 10, with higher scores indicating a stronger dependence on nicotine. Mild: 0 – 3; Moderate: 4 – 6; Severe: ≥ 7

^e^ Glover-Nilsson Smoking Behavioral Questionnaire: Possible scores range between 0 and 44, with higher scores indicating greater behavioral dependence on smoking. Mild: 0 – 12; Moderate: 12 – 22; Strong: 12 – 33; Very strong: ≥ 34

^f^ Beck Depression Inventory-II: Possible scores range between 0 and 63, with higher scores indicating greater depressive symptoms. Minimal: 0 – 13; Mild: 14 – 19; Moderate: 20 – 28; Severe: ≥ 29

^g^ Medical history was self-reported

^h^ Defined as prior use of medication for depression

^I^ Other respiratory problems include: chronic pneumonia, shortness of breath, and sleep apnea

**Supplemental Table 10:** Sensitivity analyses for point prevalence abstinence at 12, 24, and 52 weeks, adjusting for imbalances in baseline participant characteristics

|  | Nicotine E-Cigarettes + Counseling vs Counseling Alone | | | Nicotine E-Cigarettes + Counseling vs  Non-Nicotine E-Cigarettes + Counseling | | | Non-Nicotine E-Cigarettes + Counseling vs Counseling Alone | | |
| --- | --- | --- | --- | --- | --- | --- | --- | --- | --- |
| Follow-up | Crude Odds Ratio  (95% CI) | Adjusted^a^ Odds Ratio  (95% CI) | Comparison^b^ Odds Ratio  (95% CI) | Crude Odds Ratio  (95% CI) | Adjusted^a^ Odds Ratio  (95% CI) | Comparison^b^ Odds Ratio  (95% CI) | Crude Odds Ratio  (95% CI) | Adjusted^a^ Odds Ratio  (95% CI) | Comparison^b^ Odds Ratio  (95% CI) |
| Week 12 | 2.8 (1.3-5.9) | 3.0 (1.3-6.8) | 2.8 (1.3-6.2) | 1.3 (0.7-2.5) | 1.4 (0.7-2.7) | 1.4 (0.7-2.8) | 2.1 (1.0-4.5) | 2.2 (0.9-4.9) | 2.0 (0.9-4.5) |
| Week 24 | 1.7 (0.8-3.6) | 1.8 (0.8-4.1) | 1.9 (0.9-4.1) | 0.8 (0.4-1.5) | 0.8 (0.4-1.5) | 0.8 (0.4-1.6) | 2.1 (1.0-4.4) | 2.4 (1.1-5.1) | 2.2 (1.1-4.7) |
| Week 52 | 2.8 (1.4-5.8) | 2.9 (1.3-6.5) | 2.8 (1.3-6.0) | 1.3 (0.7-2.3) | 1.2 (0.6-2.3) | 1.2 (0.6-2.4) | 2.2 (1.1-4.7) | 2.4 (1.1-5.4) | 2.3 (1.1-5.1) |

Abbreviations: CI = confidence interval

^a^ Logistic regression models adjusted for all baseline characteristics for which the absolute value of the standardized difference was ≥0.1 for any pair-wise comparison (eTable 6)

^b^ Logistic regression models adjusted for all baseline characteristics for which the absolute value of the standardized difference was ≥0.1 for the following comparison:

- For Nicotine vs Counseling: Adjusted by “Self-Reported Race”, “Previously tried an e-cigarette”, “Motivation to Stop Scale score”, “Glover-Nilsson score”, “Depression”, “Diabetes”, “Hypertension”, “Asthma”, and “Other respiratory problems”
- For Nicotine vs Placebo: Adjusted by “Self-Reported Race”, “Previously attempted to quit”, “Previously tried an e-cigarette”, “Other smoker(s) at home”, “Motivation to Stop Scale score”, “Beck Depression Inventory score”, and “Hypertension”
- For Placebo vs Counseling: Adjusted by “Male”, “Self-Reported Race”, “Previously tried an e-cigarette”, “Other smoker(s) at home”, “Motivation to Stop Scale score”, “Fagerström Test score”, “Glover-Nilsson score”, “Beck Depression Inventory score”, “Diabetes”, “Respiratory problems”, and “Other respiratory problems”

**Supplemental Table 11:** Post-hoc sensitivity analysis for change in cigarettes smoked per day, adjusting for amount smoked at baseline

| Treatment Comparison | Follow-up Duration  (Weeks) | Change in Cigarettes Smoked per Day  (95% CI)* | |
| --- | --- | --- | --- |
|  |  | Crude | Adjusted for Baseline Amount Smoked |
| Nicotine vs Counseling | 1 | -6.9 (-9.1 to -4.7) | -7.2 (-9.2 to -5.1) |
| Nicotine vs Counseling | 2 | -7.6 (-10.0 to -5.1) | -7.9 (-10.1 to -5.7) |
| Nicotine vs Counseling | 4 | -7.8 (-10.1 to -5.5) | -8.1 (-10.3 to -6.0) |
| Nicotine vs Counseling | 8 | -7.3 (-9.7 to -5.0) | -7.6 (-9.8 to -5.4) |
| Nicotine vs Counseling | 12 | -5.7 (-8.0 to -3.3) | -6.0 (-8.1 to -3.8) |
| Nicotine vs Counseling | 18 | -4.5 (-7.1 to -1.9) | -4.8 (-7.2 to -2.5) |
| Nicotine vs Counseling | 24 | -5.0 (-7.4 to -2.5) | -5.2 (-7.5 to -2.9) |
| Nicotine vs Counseling | 52 | -3.9 (-6.5 to -1.4) | -4.3 (-6.6 to -2.0) |
| Nicotine vs Placebo | 1 | -0.6 (-2.9 to 1.8) | -0.9 (-2.8 to 1.0) |
| Nicotine vs Placebo | 2 | -1.5 (-4.0 to 0.9) | -1.9 (-3.9 to 0.1) |
| Nicotine vs Placebo | 4 | -2.5 (-4.9 to -0.2) | -2.9 (-4.8 to -1.0) |
| Nicotine vs Placebo | 8 | -2.6 (-5.3 to 0.1) | -3.0 (-5.3 to -0.6) |
| Nicotine vs Placebo | 12 | -2.0 (-4.7 to 0.6) | -2.4 (-4.7 to -0.2) |
| Nicotine vs Placebo | 18 | -1.6 (-4.3 to 1.1) | -2.0 (-4.3 to 0.4) |
| Nicotine vs Placebo | 24 | -1.6 (-4.3 to 1.1) | -1.9 (-4.3 to 0.4) |
| Nicotine vs Placebo | 52 | -0.2 (-3.0 to 2.6) | -0.7 (-3.1 to 1.8) |
| Placebo vs Counseling | 1 | -6.4 (-8.8 to -3.9) | -6.4 (-8.5 to -4.2) |
| Placebo vs Counseling | 2 | -6.0 (-8.6 to -3.4) | -6.0 (-8.4 to -3.7) |
| Placebo vs Counseling | 4 | -5.3 (-7.8 to -2.8) | -5.3 (-7.6 to -3.0) |
| Placebo vs Counseling | 8 | -4.8 (-7.4 to -2.1) | -4.8 (-7.2 to -2.3) |
| Placebo vs Counseling | 12 | -3.6 (-6.3 to -1.0) | -3.6 (-6.0 to -1.) |
| Placebo vs Counseling | 18 | -2.9 (-5.8 to 0.0) | -2.9 (-5.4 to -0.4) |
| Placebo vs Counseling | 24 | -3.4 (-6.0 to -0.7) | -3.4 (-5.9 to -0.9) |
| Placebo vs Counseling | 52 | -3.7 (-6.4 to -1.0) | -3.7 (-6.2 to -1.3) |

*Assuming those who were lost-to-follow-up returned to baseline smoking levels.

**Supplemental Table 12.** Post-hoc analyses for point prevalence abstinence at 12, 24, and 52 weeks, accounting for clustering by site^a^

|  | Nicotine E-Cigarettes + Counseling vs Counseling Alone | | | Nicotine E-Cigarettes + Counseling vs  Non-Nicotine E-Cigarettes + Counseling | | | Non-Nicotine E-Cigarettes + Counseling vs Counseling Alone | | |
| --- | --- | --- | --- | --- | --- | --- | --- | --- | --- |
| Follow-up | Crude Odds Ratio  (95% CI) | Adjusted^b^  Odds Ratio  (95% CI) | Comparison^c^ Odds Ratio  (95% CI) | Crude Odds Ratio  (95% CI) | Adjusted^b^  Odds Ratio  (95% CI) | Comparison^c^ Odds Ratio  (95% CI) | Crude Odds Ratio  (95% CI) | Adjusted^b^ Odds Ratio  (95% CI) | Comparison^c^ Odds Ratio  (95% CI) |
| Week 12 | 2.8 (1.3-5.9) | 3.0 (1.3-6.8) | 2.8 (1.3-6.2) | 1.3 (0.7-2.5) | 1.4 (0.7-2.7) | 1.3 (0.7-2.5) | 2.1 (1.0-4.5) | 2.2 (0.9-5.0) | 2.2 (0.9-5.0) |
| Week 24 | 1.7 (0.8-3.6) | 1.8 (0.8-4.1) | 1.9 (0.9-4.2) | 0.8 (0.4-1.5) | 0.8 (0.4-1.5) | 0.8 (0.4-1.6) | 2.1 (1.0-4.4) | 2.4 (1.1-5.1) | 2.4 (1.1-5.2) |
| Week 52 | 2.8 (1.4-5.8) | 2.9 (1.3-6.5) | 2.8 (1.3-6.0) | 1.3 (0.7-2.3) | 1.2 (0.6-2.3) | 1.3 (0.7-2.4) | 2.2 (1.1-4.7) | 2.4 (1.1-5.4) | 2.4 (1.1-5.3) |

Abbreviations: CI = confidence interval

^a^ Generalized linear mixed models included site as a random effect

^b^ Adjusted for all baseline characteristics for which the absolute value of the standardized difference was ≥0.1 for any pair-wise comparison (eTable 6)

^c^ Logistic regression models adjusted for all baseline characteristics for which the absolute value of the standardized difference was ≥0.1 for the following comparison:

- For Nicotine vs Counseling: Adjusted by “Self-Reported Race”, “Previously tried an e-cigarette”, “Motivation to Stop Scale score”, “Glover-Nilsson score”, “Depression”, “Diabetes”, “Hypertension”, “Asthma”, and “Other respiratory problems”
- For Nicotine vs Placebo: Adjusted by “Self-Reported Race”, “Previously attempted to quit”, “Previously tried an e-cigarette”, “Other smoker(s) at home”, “Motivation to Stop Scale score”, “Beck Depression Inventory score”, and “Hypertension”
- For Placebo vs Counseling: Adjusted by “Male”, “Self-Reported Race”, “Previously tried an e-cigarette”, “Other smoker(s) at home”, “Motivation to Stop Scale score”, “Fagerström Test score”, “Glover-Nilsson score”, “Beck Depression Inventory score”, “Diabetes”, “Respiratory problems”, and “Other respiratory problems”

**Supplemental Table 13:** Risk differences (95% CI) for abstinence or ≥ 50% reduction of daily cigarette consumption from baseline between treatment groups.

1. Abstinence or ≥ 50% reduction of daily cigarette consumption from baseline^a^: nicotine e-cigarettes plus counseling vs counseling alone

|  | Primary Analysis – Participants Missing Smoking Data Assumed to Have Returned to Smoking at Baseline Level | | | Sensitivity Analysis – Restricted to Participants with Self-Reported Smoking Data | | | Sensitivity Analysis – Multiple Imputation | | |
| --- | --- | --- | --- | --- | --- | --- | --- | --- | --- |
|  | Abstinence or ≥ 50% reduction of daily cigarette consumption composite | |  | Abstinence or ≥ 50% reduction of daily cigarette consumption composite | |  | Abstinence or ≥ 50% reduction of daily cigarette consumption composite | |  |
| Follow-up | Nicotine  E-Cigarettes  + Counseling | Counseling Alone | Risk Difference  (95% CI) | Nicotine  E-Cigarettes  + Counseling | Counseling Alone | Risk Difference  (95% CI) | Nicotine  E-Cigarettes  + Counseling | Counseling Alone | Risk Difference  (95% CI) |
| Week 1 | 93/128 (72.7%) | 43/121 (35.5%) | 37.1%  (25.6-48.6) | 93/119 (78.2%) | 43/94 (45.7%) | 32.4%  (19.9-44.9) | 98/128 (76.8%) | 57/121 (47.0%) | 31.2%  (19.0-43.5) |
| Week 2 | 97/128 (75.8%) | 43/121 (35.5%) | 40.2%  (28.9-51.6) | 97/119 (81.5%) | 43/87 (49.4%) | 32.1%  (19.5-44.7) | 102/128 (79.8%) | 63/121 (52.2%) | 26.0%  (13.1-39.0) |
| Week 4 | 104/128 (81.3%) | 47/121 (38.8%) | 42.4%  (31.4-53.4) | 104/119 (87.4%) | 47/83 (56.6%) | 30.8%  (18.6-43.0) | 110/128 (85.6%) | 71/121 (58.8%) | 26.9%  (12.1-41.7) |
| Week 8 | 93/128 (72.7%) | 47/121 (38.8%) | 33.8%  (22.2-45.4) | 93/112 (83.0%) | 47/79 (59.5%) | 23.5%  (10.7-36.4) | 102/128 (79.9%) | 73/121 (60.6%) | 18.9%  (5.5-32.2) |
| Week 12 | 89/128 (69.5%) | 45/121 (37.2%) | 32.3%  (20.6-44.1) | 89/113 (78.8%) | 45/81 (55.6%) | 23.2%  (10.0-36.4) | 99/128 (77.2%) | 70/121 (58.0%) | 20.8%  (6.3-35.4) |
| Week 18 | 79/128 (61.7%) | 43/121 (35.5%) | 26.2%  (14.2-38.2) | 79/106 (74.5%) | 43/77 (55.8%) | 18.7%  (4.8-32.5) | 93/128 (72.5%) | 72/121 (59.2%) | 14.4%  (1.7-27.2) |
| Week 24 | 70/128 (54.7%) | 43/121 (35.5%) | 19.2%  (7.0-31.3) | 70/106 (66.0%) | 43/70 (61.4%) | 4.6%  (-9.9 to 19.2) | 84/128 (65.3%) | 75/121 (61.6%) | 4.0%  (-9.7 to 17.6) |
| Week 52 | 65/127 (51.2%) | 37/121 (30.6%) | 20.6%  (8.7-32.6) | 65/103 (63.1%) | 37/68 (54.4%) | 8.7%  (-6.4 to 23.8) | 79/127 (61.9%) | 70/121 (58.1%) | 5.0%  (-8.5 to 18.5) |

Abbreviations: CI = confidence interval

^a^ Mean change in the number of self-reported cigarettes smoked per day in the past week

1. Abstinence or ≥ 50% reduction of daily cigarette consumption from baseline^a^: nicotine e-cigarettes plus counseling vs non-nicotine e-cigarettes plus counseling

|  | Primary Analysis – Participants Missing Smoking Data Assumed to Have Returned to Smoking at Baseline Level | | | Sensitivity Analysis – Restricted to Participants with Self-Reported Smoking Data | | | Sensitivity Analysis – Multiple Imputation | | |
| --- | --- | --- | --- | --- | --- | --- | --- | --- | --- |
|  | Abstinence or ≥ 50% reduction of daily cigarette consumption composite | |  | Abstinence or ≥ 50% reduction of daily cigarette consumption composite | |  | Abstinence or ≥ 50% reduction of daily cigarette consumption composite | |  |
| Follow-up | Nicotine  E-Cigarettes  + Counseling | Non-Nicotine  E-Cigarettes  + Counseling | Risk Difference  (95% CI) | Nicotine  E-Cigarettes  + Counseling | Non-Nicotine  E-Cigarettes  + Counseling | Risk Difference  (95% CI) | Nicotine  E-Cigarettes  + Counseling | Non-Nicotine  E-Cigarettes  + Counseling | Risk Difference  (95% CI) |
| Week 1 | 93/128 (72.7%) | 85/127 (66.9%) | 5.7%  (-5.5 to 17.0) | 93/119 (78.2%) | 85/122 (69.7%) | 8.5%  (-2.6 to 19.5) | 98/128 (76.8%) | 88/127 (69.1%) | 8.5%  (-3.3 to 20.4) |
| Week 2 | 97/128 (75.8%) | 89/127 (70.1%) | 5.7%  (-5.2 to 16.6) | 97/119 (81.5%) | 89/118 (75.4%) | 6.1%  (-4.4 to 16.5) | 102/128 (79.8%) | 94/127 (74.1%) | 4.7%  (-5.7 to 15.2) |
| Week 4 | 104/128 (81.3%) | 82/127 (64.6%) | 16.7%  (6.0-27.4) | 104/119 (87.4%) | 82/116 (70.7%) | 16.7%  (6.5-26.9) | 110/128 (85.6%) | 89/127 (70.2%) | 15.5%  (5.0-26.1) |
| Week 8 | 93/128 (72.7%) | 74/127 (58.3%) | 14.4%  (2.9-25.9) | 93/112 (83.0%) | 74/104 (71.2%) | 11.9%  (0.7-23.0) | 102/128 (79.9%) | 90/127 (71.1%) | 9.5%  (-1.9 to 20.8) |
| Week 12 | 89/128 (69.5%) | 70/127 (55.1%) | 14.4%  (2.7-26.2) | 89/113 (78.8%) | 70/102 (68.6%) | 10.1%  (-1.6 to 21.9) | 99/128 (77.2%) | 87/127 (68.5%) | 9.2%  (-2.0 to 20.3) |
| Week 18 | 79/128 (61.7%) | 69/127 (54.3%) | 7.4%  (-4.7 to 19.5) | 79/106 (74.5%) | 69/97 (71.1%) | 3.4%  (-8.9 to 15.7) | 93/128 (72.5%) | 92/127 (72.4%) | 0.8%  (-10.5 to 12.2) |
| Week 24 | 70/128 (54.7%) | 66/127 (52.0%) | 2.7%  (-9.5 to 15.0) | 70/106 (66.0%) | 66/96 (68.8%) | -2.7%  (-15.6 to 10.2) | 84/128 (78.6%) | 88/127 (69.3%) | -3.7%  (-16.9 to 9.6) |
| Week 52 | 65/127 (51.2%) | 60/127 (47.2%) | 3.9%  (-8.4% to 16.2) | 65/103 (63.1%) | 60/97 (61.9%) | 1.3%  (-12.2 to 14.7) | 79/127 (61.9%) | 79/127 (62.0%) | -0.3%  (-12.3 to 11.7) |

Abbreviations: CI = confidence interval

^a^ Mean change in the number of self-reported cigarettes smoked per day in the past week

1. Abstinence or ≥ 50% reduction of daily cigarette consumption from baseline^a^: non-nicotine e-cigarettes plus counseling vs counseling alone

|  | Primary Analysis – Participants Missing Smoking Data Assumed to Have Returned to Smoking at Baseline Level | | | Sensitivity Analysis – Restricted to Participants with Self-Reported Smoking Data | | | Sensitivity Analysis Multiple Imputation | | |
| --- | --- | --- | --- | --- | --- | --- | --- | --- | --- |
|  | Abstinence or ≥ 50% reduction of daily cigarette consumption composite | |  | Abstinence or ≥ 50% reduction of daily cigarette consumption composite | |  | Abstinence or ≥ 50% reduction of daily cigarette consumption composite | |  |
| Follow-up | Non-Nicotine  E-Cigarettes  + Counseling | Counseling Alone | Risk Difference  (95% CI) | Non-Nicotine  E-Cigarettes  + Counseling | Counseling Alone | Risk Difference  (95% CI) | Non-Nicotine  E-Cigarettes  + Counseling | Counseling Alone | Risk Difference  (95% CI) |
| Week 1 | 85/127 (66.9%) | 43/121 (35.5%) | 31.4%  (19.6-43.2) | 85/122 (69.7%) | 43/94 (45.7%) | 23.9%  (11.0-36.9) | 88/127 (69.1%) | 57/121 (47.0%) | 22.7%  (9.0-36.4) |
| Week 2 | 89/127 (70.1%) | 43/121 (35.5%) | 34.5%  (22.9-46.2) | 89/118 (75.4%) | 43/87 (49.4%) | 26.0%  (12.9-39.1) | 94/127 (74.1%) | 63/121 (52.2%) | 21.3%  (8.3-34.3) |
| Week 4 | 82/127 (64.6%) | 47/121 (38.8%) | 25.7%  (13.7-37.8) | 82/116 (70.7%) | 47/83 (56.6%) | 14.1%  (0.6-27.6) | 89/127 (70.2%) | 71/121 (58.8%) | 11.4%  (-2.4 to 25.2) |
| Week 8 | 74/127 (58.3%) | 47/121 (38.8%) | 19.4%  (7.2-31.6) | 74/104 (71.2%) | 47/79 (59.5%) | 11.7%  (-2.2 to 25.6) | 90/127 (71.1%) | 73/121 (60.6%) | 9.4%  (-3.2 to 22.1) |
| Week 12 | 70/127 (55.1%) | 45/121 (37.2%) | 17.9%  (5.7-30.1) | 70/102 (68.6%) | 45/81 (55.6%) | 13.1%  (-1.0 to 27.2) | 87/127 (68.5%) | 70/121 (58.0%) | 11.7%  (-3.4 to 26.7) |
| Week 18 | 69/127 (54.3%) | 43/121 (35.5%) | 18.8%  (6.6-31.0) | 69/97 (71.1%) | 43/77 (55.8%) | 15.3%  (1.0-29.6) | 92/127 (72.4%) | 72/121 (59.2%) | 13.6%  (1.1-26.1) |
| Week 24 | 66/127 (52.0%) | 43/121 (35.5%) | 16.4%  (4.3-28.6) | 66/96 (68.8%) | 43/70 (61.4%) | 7.3%  (-7.4 to 22.0) | 88/127 (69.3%) | 75/121 (61.6%) | 7.6%  (-8.6 to 23.9) |
| Week 52 | 60/127 (47.2%) | 37/121 (30.6%) | 16.7%  (4.7-28.6) | 60/97 (61.9%) | 37/68 (54.4%) | 7.4%  (-7.8 to 22.7) | 79/127 (62.0%) | 70/121 (58.1%) | 5.3%  (-7.7 to 18.4) |

Abbreviations: CI = confidence interval

^a^ Mean change in the number of self-reported cigarettes smoked per day in the past week

**Supplemental Table 14:** Risk differences (95% CI) for prolonged continuous smoking abstinence between treatment groups

1. Prolonged^a^ continuous abstinence^b^: nicotine e-cigarettes plus counseling vs counseling alone

|  | Primary Analysis – Participants Missing Smoking Data Assumed to Have Returned to Smoking at Baseline Level | | | Sensitivity Analysis – Restricted to Participants with Self-Reported Smoking Data | | | Sensitivity Analysis – Multiple Imputation | | |
| --- | --- | --- | --- | --- | --- | --- | --- | --- | --- |
|  | Abstinence | |  | Abstinence | |  | Abstinence | |  |
| Follow-up | Nicotine  E-Cigarettes + Counseling | Counseling Alone | Risk Difference  (95% CI) | Nicotine  E-Cigarettes  + Counseling | Counseling Alone | Risk Difference  (95% CI) | Nicotine  E-Cigarettes  + Counseling | Counseling Alone | Risk Difference  (95% CI) |
| Week 4 | 27/128 (21.1%) | 10/121 (8.3%) | 12.8%  (4.2-21.4) | 21/96 (21.9%) | 6/58 (10.3%) | 11.5%  (0.9-22.9) | 28/128 (22.2%) | 16/121(12.9%) | 9.3%  (-1.4 to 20) |
| Week 8 | 23/128 (18.0%) | 6/121 (5.0%) | 13.0%  (5.3-20.7) | 18/96 (18.8%) | 5/58 (8.6%) | 10.1%  (-0.5 to 20.8) | 23/128 (18.3%) | 8/121 (6.9%) | 11.3%  (2.2-20.5) |
| Week 12 | 17/128 (13.3%) | 5/121 (4.1%) | 9.2%  (2.3-16.0) | 13/96 (13.5%) | 4/58 (6.9%) | 6.7%  (-2.8 to 16.1) | 17/128 (13.3%) | 6/121 (5.1%) | 8.2%  (0.6-15.7) |
| Week 18 | 13/128 (10.2%) | 4/121 (3.3%) | 6.9%  (0.7-13.0) | 12/96 (12.5%) | 3/58 (5.2%) | 7.3%  (-1.4 to 16.1) | 14/128 (10.8%) | 4/121 (3.5%) | 7.3%  (0.9-13.7) |
| Week 24 | 10/128 (7.8%) | 4/121 (3.3%) | 4.5%  (-1.1 to 10.1) | 10/96 (10.4%) | 3/58 (5.2%) | 5.2%  (-3.1 to 13.6) | 11/128 (8.8%) | 4/121 (3.3%) | 5.4%  (-0.5 to 11.4) |
| Week 52 | 8/127 (6.3%) | 2/121 (1.7%) | 4.7%  (-0.2 to 9.4) | 8/96 (8.3%) | 2/58 (3.4%) | 4.9%  (-2.4 to 12.1) | 8/127 (6.5%) | 2/121 (2.0%) | 4.5%  (-0.6 to 9.6) |

Abbreviations: CI = confidence interval

^a^ Prolonged abstinence, defined as self-reported abstinence all follow-up visits after an initial 4-week grace period

^b^ Participants were considered abstinent if they abstained from smoking in the 7 days before the visit through a self-report of 0 cigarettes smoked/day, at all follow-ups since week 4, with a carbon monoxide monitor reading ≤10 ppm (available for 78%, 82%, 78%, and 86% of continuously self-reported abstinent participants at weeks 4, 12, 24, and 52, respectively)

1. Prolonged^a^ continuous abstinence^b^: nicotine e-cigarettes plus counseling vs non-nicotine e-cigarettes plus counseling

|  | Primary Analysis – Participants Missing Smoking Data Assumed to Have Returned to Smoking at Baseline Level | | | Sensitivity Analysis, Restricted to Participants with Self-Reported Smoking Data | | | Sensitivity Analysis, Multiple Imputation | | |
| --- | --- | --- | --- | --- | --- | --- | --- | --- | --- |
|  | Abstinence | |  | Abstinence | |  | Abstinence | |  |
| Follow-up | Nicotine  E-Cigarettes + Counseling | Non-Nicotine  E-Cigarettes + Counseling | Risk Difference  (95% CI) | Nicotine  E-Cigarettes  + Counseling | Non-Nicotine  E-Cigarettes + Counseling | Risk Difference  (95% CI) | Non-Nicotine  E-Cigarettes + Counseling | Counseling Alone | Risk Difference  (95% CI) |
| Week 4 | 27/128 (21.1%) | 16/127 (12.6%) | 8.5%  (-0.6 to 17.6) | 21/96 (21.9%) | 13/86 (15.1%) | 6.8%  (-4.4 to 18.0) | 28/128 (22.2%) | 17/127 (13.4%) | 8.8%  (-1.1 to 18.7) |
| Week 8 | 23/128 (18.0%) | 10/127 (7.9%) | 10.1%  (2.0-18.2) | 18/96 (18.8%) | 8/86 (9.3%) | 9.4%  (-0.5 to 19.4) | 23/128 (18.3%) | 11/127 (8.3%) | 9.9%  (1.7-18.2) |
| Week 12 | 17/128 (13.3%) | 9/127 (7.1%) | 6.2%  (-1.2 to 13.6) | 13/96 (13.5%) | 8/86 (9.3%) | 4.2%  (-4.9 to 13.4) | 17/128 (13.3%) | 9/127 (7.2%) | 6.0%  (-1.4 to 13.5) |
| Week 18 | 13/128 (10.2%) | 7/127 (5.5%) | 4.6%  (-1.9 to 11.2) | 12/96 (12.5%) | 7/86 (8.1%) | 4.4%  (-4.4 to 13.1) | 14/128 (10.8%) | 8/127 (6.0%) | 4.8%  (-2.1 to 11.7) |
| Week 24 | 10/128 (7.8%) | 7/127 (5.5%) | 2.3%  (-3.8 to 8.4) | 10/96 (10.4%) | 7/86 (8.1%) | 2.3%  (-6.1 to 10.7) | 11/128 (8.8%) | 7/127 (5.5%) | 3.2%  (-2.3 to 9.6) |
| Week 52 | 8/127 (6.3%) | 7/127 (5.5%) | 0.8%  (-5.0 to 6.6) | 8/96 (8.3%) | 7/86 (8.1%) | 0.3%  (-7.8 to 8.2) | 8/127 (6.5%) | 7/127 (5.5%) | 0.9%  (-4.9 to 6.8) |

Abbreviations: CI = confidence interval

^a^ Prolonged abstinence, defined as self-reported abstinence all follow-up visits after an initial 4-week grace period

^b^ Participants were considered abstinent if they abstained from smoking in the 7 days before the visit through a self-report of 0 cigarettes smoked/day, at all follow-ups since week 4, with a carbon monoxide monitor reading ≤10 ppm (available for 78%, 82%, 78%, and 86% of continuously self-reported abstinent participants at weeks 4, 12, 24, and 52, respectively)

1. Prolonged^a^ continuous abstinence^b^: non-nicotine e-cigarettes plus counseling vs counseling alone

|  | Primary Analysis – Participants Missing Smoking Data Assumed to Have Returned to Smoking at Baseline Level | | | Sensitivity Analysis – Restricted to Participants with Self-Reported Smoking Data | | | Sensitivity Analysis – Multiple Imputation | | |
| --- | --- | --- | --- | --- | --- | --- | --- | --- | --- |
|  | Abstinence | |  | Abstinence | |  | Abstinence | |  |
| Follow-up | Non-Nicotine  E-Cigarettes + Counseling | Counseling Alone | Risk Difference  (95% CI) | Non-Nicotine  E-Cigarettes  + Counseling | Counseling Alone | Risk Difference  (95% CI) | Non-Nicotine  E-Cigarettes  + Counseling | Counseling Alone | Risk Difference  (95% CI) |
| Week 4 | 16/127 (12.6%) | 10/121 (8.3%) | 4.3%  (-3.2 to 11.9) | 13/86 (15.1%) | 6/58 (10.3%) | 4.8%  (-6.1 to 15.7) | 17/127 (13.4%) | 16/121 (12.9%) | 0.5%  (-9.0 to 10.0) |
| Week 8 | 10/127 (7.9%) | 6/121 (5.0%) | 2.9%  (-3.2 to 9.0) | 8/86 (9.3%) | 5/58 (8.6%) | 0.7%  (-8.8 to 10.2) | 11/127 (8.3%) | 8/121 (6.9%) | 1.4%  (-6.3 to 9.1) |
| Week 12 | 9/127 (7.1%) | 5/121 (4.1%) | 3.0%  (-2.8 to 8.7) | 8/86 (9.3%) | 4/58 (6.9%) | 2.4%  (-6.5 to 11.4) | 9/127 (7.2%) | 6/121 (5.1%) | 2.1%  (-4.2 to 8.5) |
| Week 18 | 7/127 (5.5%) | 4/121 (3.3%) | 2.2%  (-2.9% to 7.3) | 7/86 (8.1%) | 3/58 (5.2%) | 3.0%  (-5.1 to 11.1) | 8/127 (6.0%) | 4/121 (3.5%) | 2.5%  (-2.8 to 7.8) |
| Week 24 | 7/127 (5.5%) | 4/121 (3.3%) | 2.2%  (-2.9 to 7.3) | 7/86 (8.1%) | 3/58 (5.2%) | 3.0%  (-5.1 to 11.1) | 7/127 (5.5%) | 4/121 (3.3%) | 2.2%  (-2.9 to 7.3) |
| Week 52 | 7/127 (5.5%) | 2/121 (1.7%) | 3.9%  (-0.7 to 8.4) | 7/86 (8.1%) | 2/58 (3.4%) | 4.7%  (-2.8 to 12.1) | 7/127 (5.5%) | 2/121 (2.0%) | 3.5%  (-1.2 to 8.3) |

Abbreviations: CI = confidence interval

^a^ Prolonged abstinence, defined as self-reported abstinence all follow-up visits after an initial 4-week grace period

^b^ Participants were considered abstinent if they abstained from smoking in the 7 days before the visit through a self-report of 0 cigarettes smoked/day, at all follow-ups since week 4, with a carbon monoxide monitor reading ≤10 ppm (available for 78%, 82%, 78%, and 86% of continuously self-reported abstinent participants at weeks 4, 12, 24, and 52, respectively)

**Supplemental Table 15:** Regression analyses for point prevalence abstinence at 12, 24, and 52 weeks, adjusted for use of non-study smoking cessation aids and use of abstinence aids^a^

|  | Nicotine E-Cigarettes + Counseling vs Counseling Alone | | | | Nicotine E-Cigarettes + Counseling vs Non-Nicotine E-Cigarettes + Counseling | | | | Non-Nicotine E-Cigarettes + Counseling vs Counseling Alone | | | |
| --- | --- | --- | --- | --- | --- | --- | --- | --- | --- | --- | --- | --- |
| Follow-up | Crude Odds Ratio  (95% CI) | Adjusted^b^ Odds Ratio  (95% CI) | Adjusted^c^ Odds Ratio  (95% CI) | Adjusted^d^ Odds Ratio (95% CI) | Crude Odds Ratio  (95% CI) | Adjusted^b^ Odds Ratio  (95% CI) | Adjusted^c^ Odds Ratio  (95% CI) | Adjusted^d^ Odds Ratio (95% CI) | Crude Odds Ratio  (95% CI) | Adjusted^b^ Odds Ratio  (95% CI) | Adjusted^c^ Odds Ratio  (95% CI) | Adjusted^d^ Odds Ratio (95% CI) |
| Week 12 | 2.8  (1.3-5.9) | 2.2  (1.0-5.1) | 2.2  (1.0-4.8) | 2.0  (0.9-4.4) | 1.3  (0.7-2.5) | 1.2  (0.6-2.2) | 1.3  (0.7-2.4) | 1.2  (0.7-2.3) | 2.1  (1.0-4.5) | 1.9  (0.8-4.4) | 1.8  (0.8-3.9) | 1.6  (0.7-3.6) |
| Week 24 | 1.7  (0.8-3.6) | 1.4  (0.6-3.3) | 1.6  (0.8-3.3) | 1.3  (0.6-2.7) | 0.8  (0.4-1.5) | 0.6  (0.3-1.3) | 0.8  (0.4-1.5) | 0.7  (0.4-1.4) | 2.1  (1.0-4.4) | 2.2  (0.9-5.0) | 2.0  (1.0-4.1) | 1.8  (0.8-3.7) |
| Week 52 | 2.8  (1.4-5.8) | 2.1  (0.9-4.5) | 2.8  (1.4-5.8) | 2.0  (0.9-4.2) | 1.3  (0.7-2.3) | 1.2  (0.6-2.3) | 1.3  (0.7-2.4) | 1.1  (0.6-2.0) | 2.2  (1.1-4.7) | 1.7  (0.8-3.9) | 2.1  (1.0-4.5) | 1.8  (0.8-3.9) |

Abbreviations: CI = confidence interval

^a^ Adjusted for use of non-study smoking cessation aids and use of abstinence aids

^b^ Participants excluded from analysis if missing data on use of abstinence aids

^c^ Participants assumed to have used abstinence aids if missing data on use of abstinence aids

^d^ Participants with missing data on use of abstinence aids grouped as a sub-category (yes/no/missing)

**Supplemental Table 16:** Reasons for early study participation termination

1. Reasons for early study termination that occurred between baseline to 12 weeks follow-up by treatment group

|  | Nicotine  E-Cigarettes + Counseling | Non-Nicotine  E-Cigarettes + Counseling | Counseling Alone |
| --- | --- | --- | --- |
| Standardized Study Reason | | | |
| Desire of participant* or family | 9 | 9 | 29 |
| Side effect | 1 | 3 | 0 |
| Participant too ill | 0 | 0 | 1 |
| Desire of physician | 1^a^ | 0 | 0 |
| SAE | 0 | 0 | 0 |
| Death | 0 | 0 | 0 |
| Other | 1^b^ | 0 | 0 |
| *Reason Given by the Participant | | | |
| Personal reasons | 4 | 0 | 0 |
| No reason given | 0 | 0 | 6 |
| Randomized to control group | 0 | 0 | 14 |
| Treatment not working | 3 | 3 | 2 |
| No longer wants to quit | 1 | 3 | 4 |
| Wants to try a different cessation aid | 0 | 2 | 3 |
| Worried of possible health effects of treatment | 0 | 1 | 0 |

Abbreviations: SAE = serious adverse event

^a^ Participant wanted to break randomization code

^b^ Participant moved

1. Reasons for early study termination that occurred between 12 to 52 weeks follow-up by treatment group

|  | Nicotine  E-Cigarettes + Counseling | Non-Nicotine  E-Cigarettes + Counseling | Counseling Alone |
| --- | --- | --- | --- |
| Standardized Study Reason | | | |
| Desire of participant* or family | 5 | 5 | 4 |
| Side effect | 0 | 0 | 0 |
| Participant too ill | 0 | 0 | 0 |
| Desire of physician | 0 | 0 | 0 |
| SAE | 0 | 1^a^ | 0 |
| Death | 1 | 0 | 0 |
| Other | 0 | 0 | 0 |
| *Reason Given by the Participant | | | |
| Personal reasons | 1 | 0 | 0 |
| No reason given | 0 | 3 | 1 |
| No longer interested in participating | 3 | 2 | 0 |
| Treatment not working | 0 | 0 | 1 |
| No longer wants to quit | 1 | 0 | 1 |
| Wants to try a different cessation aid | 0 | 0 | 1 |

Abbreviations: SAE = serious adverse event

^a^ The SAE was not related to study treatment

1. Overall reasons for early study termination

|  | Nicotine  E-Cigarettes + Counseling | Non-Nicotine  E-Cigarettes + Counseling | Counseling Alone |
| --- | --- | --- | --- |
| Standardized study reason | | | |
| Desire of participant* or family | 14 | 14 | 33 |
| Side effect | 1 | 3 | 0 |
| Participant too ill | 0 | 0 | 1 |
| Desire of physician | 1^a^ | 0 | 0 |
| SAE | 0 | 1^b^ | 0 |
| Death | 1 | 0 | 0 |
| Other | 1^c^ | 0 | 0 |
| *Reason given by the participant | | | |
| Personal reasons | 5 | 0 | 0 |
| No reason given | 0 | 3 | 7 |
| Randomized to control group | 0 | 0 | 14 |
| Treatment not working | 3 | 3 | 3 |
| No longer wants to quit | 2 | 3 | 5 |
| Wants to try a different cessation aid | 0 | 2 | 4 |
| No longer interested in participating | 3 | 2 | 0 |
| Worried of possible health effects of treatment | 0 | 1 | 0 |

Abbreviations: SAE = serious adverse event

^a^ Participant wanted to break randomization code

^b^ The SAE was not related to study treatment

^c^ Participant moved

**Supplemental Figure 1.** E-cigarette used in the E3 Trial

**
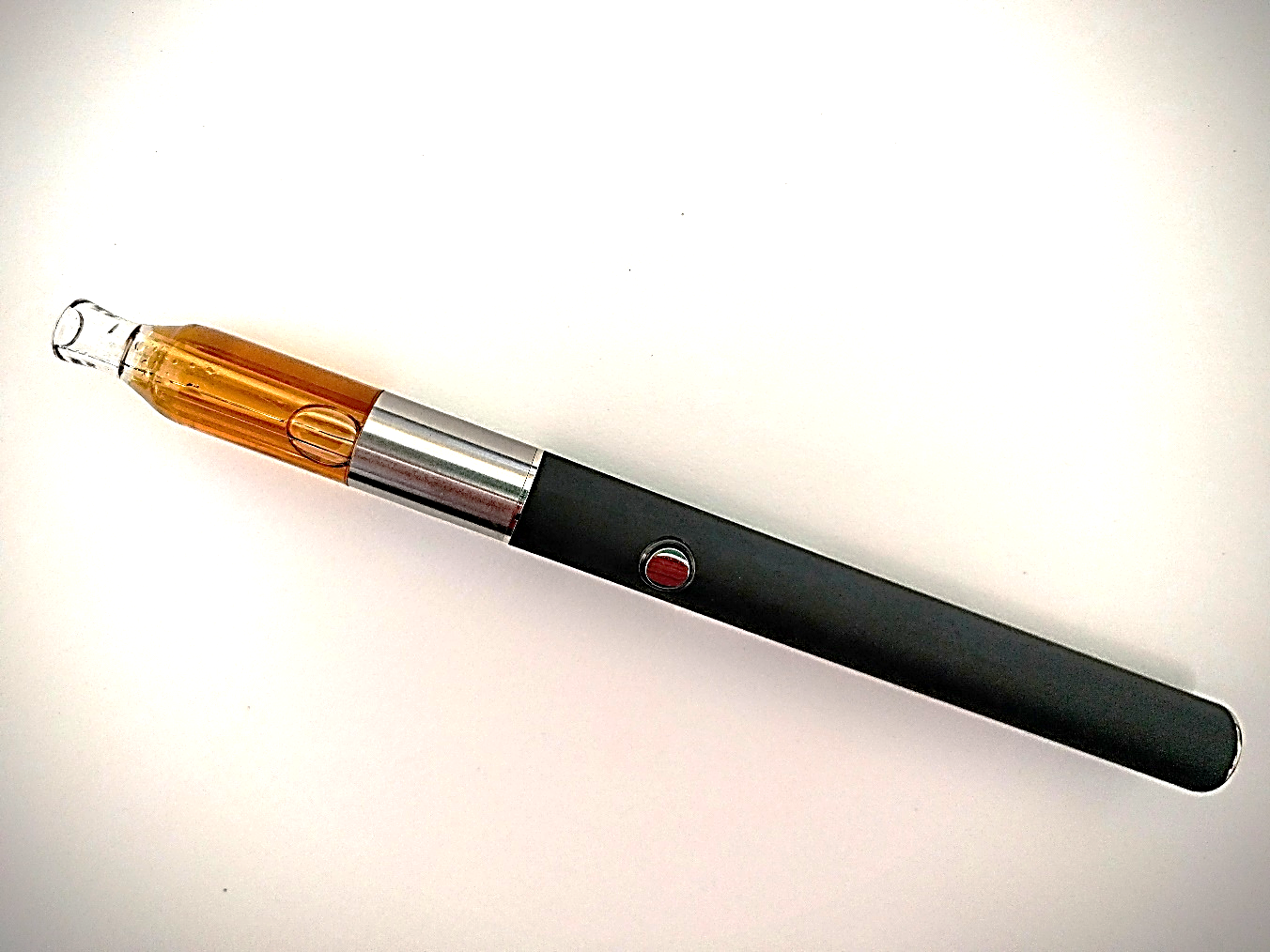
**

Nicotine and non-nicotine e-cigarettes used in the E3 Trial were produced by NJOY Inc. (Scottsdale, AZ) specifically for use in clinical studies

Device and e-liquid characteristics can be found here: <https://www.drugabuse.gov/research/research-data-measures-resources/nida-drug-supply-program/supplemental-information-nida-e-cig>

**Supplemental Figure 2.** Smoking abstinence and reduction by treatment group in the E3 Trial


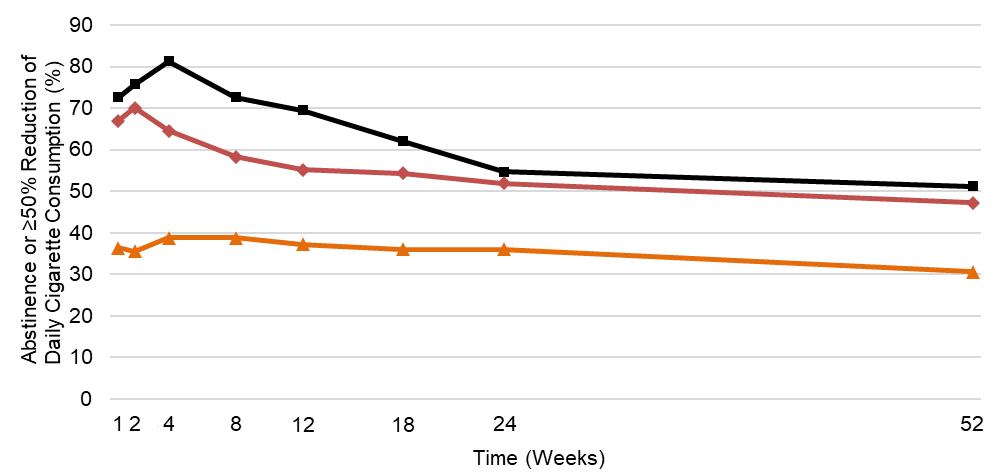


Nicotine e-cigarettes + counseling

Non-nicotine e-cigarettes + counseling

Counseling alone

*Abstinence or ≥50% reduction of daily cigarette consumption from baseline composite

*Abstinence or ≥50% reduction of daily cigarette consumption from baseline composite

**Supplemental Figure 3.** Risk differences for 7-day point prevalence smoking abstinence at 52 weeks between treatment groups abstinence by baseline characteristics

1. Nicotine e-cigarettes with individual counseling vs non-nicotine e-cigarettes with individual counseling


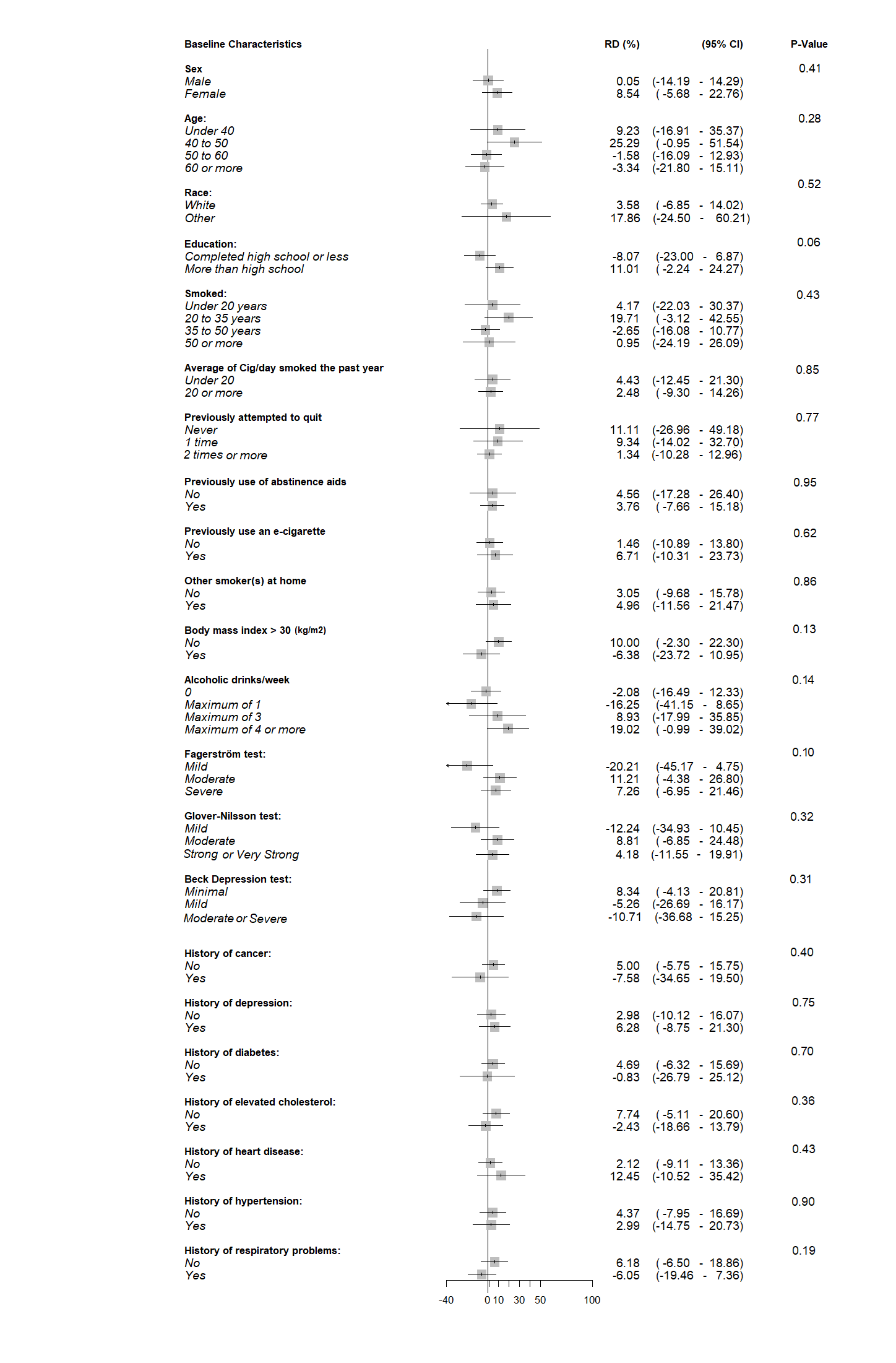


1. Non-nicotine e-cigarettes with individual counseling vs individual counseling alone


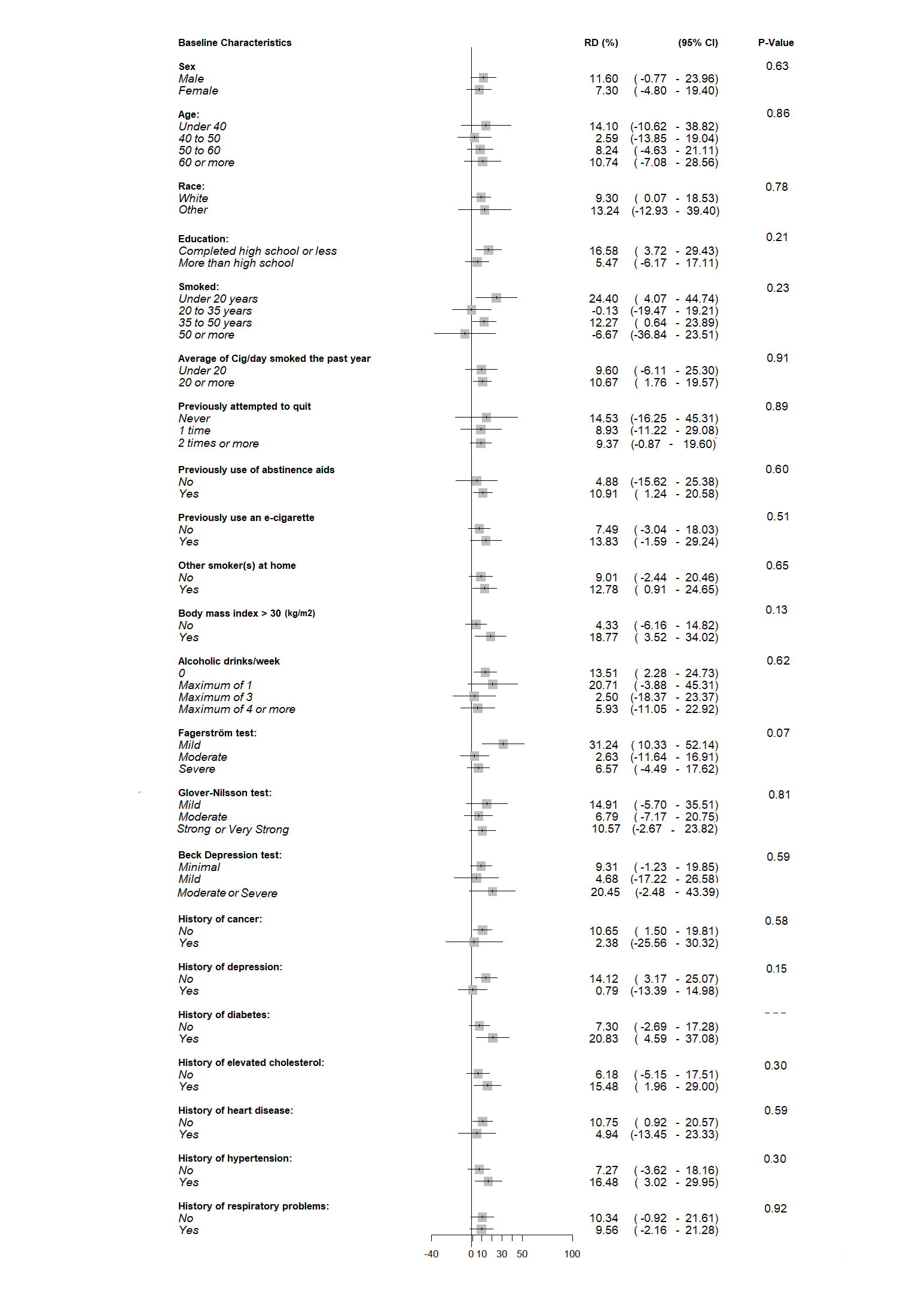


**References**

1. Frangakis C, Rubin D. Addressing complications of intention-to-treat analysis in the combined presence of all-or-none treatment-noncompliance and subsequent missing outcomes. Biometrika. 1999;86(2):365-79.
